# Functional heterogeneity and health impacts of oral bacterial ectopic colonization

**DOI:** 10.3389/fmicb.2026.1915341

**Published:** 2026-09-25

**Authors:** Kaiyang Xue, Mengzhen Ji, Ling Zou

**Affiliations:** 1 State Key Laboratory of Oral Diseases, National Center for Stomatology, National Clinical Research Center for Oral Diseases, Department of Conservative Dentistry and Endodontics, West China Hospital of Stomatology, Sichuan University, Chengdu, China; 2 Centre for Host-Microbiome Interactions, Faculty of Dentistry, Oral & Craniofacial Sciences, King’s College London, London, United Kingdom

**Keywords:** ectopic colonization, metabolic adaptation, oral microbiota, oral-intestinal-hepatic axis, systemic diseases

## Abstract

The oral cavity is the second most colonized anatomical site after the intestinal tract, and due to its unique and diverse microbial community, it constitutes a distinct biome. The oral microbiota is an integral part of the human microbiome ecosystem, maintaining the balance of the oral microecology. However, when this balance of the oral microecology is disrupted, oral bacteria not only cause oral diseases but also spread to other parts of the body through various pathways. These oral bacteria colonizing outside the oral cavity may exhibit different functional roles from those in the oral cavity, thereby contributing to or exacerbating pathological processes in distant organs, such as experimental colitis, hepatic steatosis, and opportunistic bacteremia. By analyzing compositional changes in the oral and gut microbiota, the risk of certain diseases can be predicted at an early stage, and timely interventions can be implemented. This review summarizes the colonization and disease-causing effects of several major oral bacteria (*Streptococcus, Veillonella, Prevotella, Lactobacillus, Helicobacter pylori, Fusobacterium nucleatum, Porphyromonas gingivalis, Tannerella forsythia*) in the oral cavity and other sites. To provide conceptual rigor, we explicitly distinguish among passive ectopic detection, stable ectopic colonization, and functionally active pathogenicity. Furthermore, we highlight how ectopic oral bacteria undergo context-dependent metabolic adaptations in new niches, rather than simply replicating their oral profiles.

## 1 Introduction

The oral microbiota is one of the most diverse and unique microbial communities in the human body, and also a vital component of the human microbiome, encompassing bacteria, fungi, viruses, etc. (Baker et al., 2024). Currently, the number of bacterial species cataloged in the Human Oral Microbiome Database (HOMD) is approaching 800. The maintenance of oral microbiota homeostasis is a crucial component of both oral health and overall systemic health. As the “gateway” to the digestive tract, the oral cavity has an inherent connection with other digestive organs and can interact with them at multiple levels (Baker et al., 2024; Senthil Kumar et al., 2024; Sedghi et al., 2021). Under physiological conditions, the oral microbiota, through synergistic interaction with the host immune system, maintains microecological balance and prevents pathogen invasion, serving as an important part of the body’s immune defense (Wade, 2013; Cyr et al., 2020). As an open ecosystem, changes in the abundance and diversity of the oral microbiota can not only directly affect local health, but also indirectly impact a variety of systemic diseases—including coronary atherosclerotic heart disease, diabetes mellitus, rheumatoid arthritis, inflammatory bowel disease (IBD), malnutrition, preterm birth, neurological diseases, liver diseases, lung diseases, and colon cancer—through pathways such as inflammatory responses, immune system dysregulation, bacterial translocation, and diffusion of metabolites. To a certain extent, these changes can reflect the health status of the host (Nabavi-Rad et al., 2022; Owoyele and Malekzadeh, 2022; Fan et al., 2023; Liu et al., 2023; Ma et al., 2023; Pan and Jiao, 2025). In recent years, significant progress has been made in research on the relationship between the oral microbiota and systemic diseases, and it has shown broad application prospects in the diagnosis, treatment, and prognosis evaluation of some diseases (Guo et al., 2021; Luo et al., 2024; Ye et al., 2025).

It is important to note that while the oral cavity and the gut are interconnected, they are not functionally interchangeable niches. Recent multi-omics studies have demonstrated that the oral cavity and gut maintain distinct compositional and functional signatures. Oral-derived bacteria colonizing the gut do not simply reproduce their oral functional profiles; rather, they acquire context-dependent metabolic and immunological functions adapted to the intestinal environment (Baima et al., 2025). Different from the previous disease-oriented classification models, this review takes common oral bacterial species as the core classification dimension and focuses on the functional heterogeneity of the bacteria themselves. A central limitation in current microbiome research is the frequent conflation of correlation with causation. Throughout this review, we explicitly differentiate between passive translocation or ectopic detection (the mere detection of bacterial DNA via sequencing without evidence of viability), stable ectopic colonization, and functionally active colonization with demonstrated host impact.

To cover a diverse spectrum of colonization behaviors, we selected 8 representative taxa. These include well-established core oral pathogens and commensals (*Streptococcus*, *Veillonella*, *Prevotella*, *Fusobacterium nucleatum*, *Porphyromonas gingivalis*, *Tannerella forsythia*) alongside more transient or debated colonizers (*Lactobacillus*, *Helicobacter pylori*). While taxa such as *H. pylori* are predominantly gut- or gastric-associated and their detection in the oral cavity may represent transient or secondary events, evaluating their bidirectional translocation offers valuable insights into the complexity of mucosal microbial continuity. By critically addressing the limitations of current evidence—such as the reliance on compositional 16S rRNA data, the lack of longitudinal causality, and the artificial permissiveness of animal models—this review aims to provide a more nuanced perspective. Ultimately, this approach will help mitigate the risk of premature clinical extrapolation and offer clearer directions for the development of reproducible, multi-cohort validated biomarkers and therapeutics.

## 2 Literature search strategy and evidentiary framework

To provide a comprehensive conceptual synthesis of oral bacterial ectopic colonization and its systemic implications, this article was designed as a narrative review following the SANRA (Scale for the Assessment of Narrative Review Articles) reporting guidelines. We employed a concept-driven, purposive literature search across four major electronic databases from inception to January 2026: PubMed/MEDLINE, Embase, Web of Science Core Collection, and Google Scholar, restricted to peer-reviewed English-language publications. Searches were executed across Title, Abstract, MeSH terms, and Emtree subject headings [tiab/mesh/exp] to maximize retrieval sensitivity.

The search architecture combined three core thematic domains using Boolean AND logic:

Domain 1 (Oral Taxa): Descriptors of oral microecology alongside the eight focused bacterial taxa (“Streptococcus,” “Veillonella,” “Prevotella,” “Lactobacillus,” “*Helicobacter pylori*,” “*Fusobacterium nucleatum*,” “*Porphyromonas gingivalis*,” and “*Tannerella forsythia*”);

Domain 1 (Oral Taxa): Descriptors of oral microecology alongside the eight focused bacterial taxa (“Streptococcus,” “Veillonella,” “Prevotella,” “Lactobacillus” [including reclassified taxa such as “Lacticaseibacillus” and “Lactiplantibacillus”], “*Helicobacter pylori*,” “*Fusobacterium nucleatum*,” “*Porphyromonas gingivalis*,” and “*Tannerella forsythia*”);

Domain 2 (Translocation & Niche Dynamics): Controlled terms and keywords capturing dissemination, mucosal barrier breach, and metabolic rewiring (e.g., “ectopic colonization,” “bacterial translocation,” “oral-gut axis,” “oral-gut-liver axis,” “bloodstream dissemination,” “anaerobic respiration,” “metabolicadaptation”);

Domain 3 (Target Pathologies & Systemic Outcomes): Specific MeSH and free-text terms covering multi-organ pathologies, including cardiovascular (“infective endocarditis,” “atherosclerosis,” “stroke”), gastrointestinal and hepatic (“gastritis,” “colitis,” “inflammatory bowel disease”, “colorectal neoplasms”, “MASLD/NAFLD”), respiratory (“pneumonia”, “allergic rhinitis”, “asthma”), neurological (“neuroinflammation”, “cognitive decline”, “Parkinson’s disease”), obstetric (“preterm birth”, “intrauterine infection”), and metabolic conditions (“insulin resistance”, “childhood stunting”).

Since this narrative review aimed at conceptual synthesis rather than exhaustive epidemiological meta-analysis, study selection was conducted purposively based on explicit predefined criteria. We prioritized peer-reviewed original research offering: (i) paired oral–extraoral strain-level metagenomic tracking, (ii) defined molecular/cellular mechanisms in *in vitro* or isogenic mutant models, (iii) causal interventional validation in gnotobiotic/conventional animal systems, or (iv) prospective human clinical cohort associations. Localized oral studies lacking extra-oral evaluations, non-peer-reviewed preprints, conference abstracts, and non-English articles were excluded. Title/abstract screening, full-text evaluation, and data extraction were conducted independently by two reviewers (K. X. and M. J.), with discrepancies resolved through consensus discussion mediated by a senior author (L. Z.). Extracted evidence was categorized into our multidimensional matrix according to viability status (culture vs. DNA-only), mechanistic depth, and translational clinical relevance. Full executable database-specific search strings and the literature appraisal protocol are provided in Supplementary File 1.

## 3 Role of common oral bacteria in and beyond the oral cavity

As mentioned before, the involvement of common oral bacteria in both oral and systemic diseases is increasingly recognized as crucial. This review highlights the functions of major oral bacteria (*Streptococcus*, *Veillonella*, *Prevotella, Lactobacillus*, *Helicobacter pylori*, *Fusobacterium nucleatum*, *Porphyromonas gingivalis*, *Tannerella forsythia*) within and beyond the oral cavity (Table 1). Additionally, it explores the relevance of the oral-gut-liver axis in mediating systemic effects linked to oral microbiota. These specific eight taxa were selected because they represent a diverse cross-section of the oral microbiome—ranging from early colonizers and commensals (e.g., *Streptococcus*) to established keystone pathogens (e.g., *P. gingivalis*) and opportunistic pathobionts—and possess the most robust, albeit sometimes debated, body of evidence regarding their ectopic colonization and systemic impacts.

**Table 1:** Evidence-based evaluation of oral bacterial ectopic colonization across selected systemic conditions.

Taxon	Disease/homeostasis context	Colonization site	Proposed mechanisms	Ectopic colonization	Evidence of oral origin	Evidence basis	Limitations
*Streptococcus salivarius*	Allergic Rhinitis (AR) Exacerbation	Nasal cavity	Adheres to nasal epithelial cells by binding to Mucin5AC protein; upregulates inflammation-related genes (e.g., IL-1β, IL-6, TNF-α; Miao et al., 2023).	Active Pathogenicity	Probable (genomic / anatomical contiguity)	Viability & State: Culture-proven Mechanistic Depth: Receptor-ligand binding (Mucin5AC adherence) Interventional Validation: Murine intervention (*in vivo* AR mouse model) Human Evidence: Cross-sectional (nasal cohort 16S rRNA profiling; Miao et al., 2023)	Rodent nasal mucosa differs immunologically from humans; clinical efficacy of targeted intervention lacks large-scale human validation.
	Childhood Malnutrition & Stunting	Intestine (Small intestine /Duodenum)	Secretes low-molecular-weight products that reduce intestinal lipid absorption, disrupt the epithelial barrier, and induce small intestinal inflammation (Vonaesch et al., 2022).	Active Pathogenicity	Confirmed (paired strain-level metagenomics)	Viability & State: Culture-proven (duodenal aspirates) Mechanistic Depth: *In vitro* assay (enterocyte lipid transport inhibition) Interventional Validation: Gnotobiotic animal model (germ-free mice reproducing stunting) Human Evidence: Prospective cohort (Afribiota pediatric clinical study; Vonaesch et al., 2022)	The precise molecular identities of the secreted low-molecular-weight products and their direct host receptors are still being fully characterized.
*Streptococcus anginosus*	Gastric Cancer Progression & Tumorigenesis	Gastric mucosa	In murine and cellular models, surface lipoprotein TmpC directly engages ANXA2 to trigger MAPK signaling, while bacterial MetE-driven methionine synthesis mechanistically stimulates tumor proliferation *in vitro* and in mice (Zhou et al., 2026).	Active Pathogenicity	Probable (genomic / shared oral-gastric lineage)	Viability & State: Active metabolic adaptation (methionine biosynthesis) Mechanistic Depth: Isogenic mutant (KO/rescue) ( ΔmetE knockout) & Receptor-ligand binding (TmpC-ANXA2 direct binding) Interventional Validation: Gnotobiotic animal model (germ-free & conventional mice reproducing gastric dysplasia & cancer) Human Evidence: Prospective cohort / Cross-sectional (significant mucosal enrichment in clinical GC cohorts; Fu et al., 2024; Zhou et al., 2026)	Although animal data are highly robust, the synergistic pathogenic interaction with concurrent *H. pylori* infection in humans requires further clinical longitudinal studies.
*Streptococcus sanguinis* (Strain SK36)	Infective Endocarditis (IE)	Bloodstream / Heart valves	Enters bloodstream via transient bacteremia; utilizes Type IV Pili (PilB/PilT/PilC) and surface adhesins to bind platelets and adhere to vascular endothelial cells, initiating vegetation formation (Martini et al., 2021; Raynaud et al., 2021).	Active Pathogenicity	Confirmed (paired clinical oral & cardiac valve isolates)	Viability & State: Culture-proven (persistent cardiac vegetations) Mechanistic Depth: Isogenic mutant (KO/rescue) (isogenic pilus mutants) & Receptor-ligand binding (platelet binding assays) Interventional Validation: Murine intervention (rabbit & mouse experimental infective endocarditis models) Human Evidence: Cross-sectional (clinical IE isolate genomic profiling & diagnostic blood cultures; Baker et al., 2019; Martini et al., 2021; Raynaud et al., 2021)	Although its adherence and pilus genetics are fully mapped, prevention efficacy in clinical populations (e.g., antibiotic prophylaxis) remains a subject of ongoing epidemiological debate.
*Veillonella parvula* (Strain DSM 2008 / SK347)	Ectopic Intestinal Colonization (Colitis)	Gastrointestinal tract (Intestine)	Undergoes a metabolic shift from fermentation to anaerobic nitrate respiration via the nar gene cluster (specifically *narG*), using microenvironmental nitrite for rapid proliferation (Rojas-Tapias et al., 2022).	Stable Colonization	Probable (oral-to-gut translocation)	Viability & State: Active metabolic adaptation (nitrate respiration switch) Mechanistic Depth: Isogenic mutant (KO/rescue) (isogenic ΔnarG mutant loses colonization fitness) Interventional Validation: Murine intervention (DSS colitis mouse models & host $\mathrm{iNO}S^{-/-}$ knockout validation) Human Evidence: Cross-sectional (enrichment in IBD patient metagenomic cohorts; Rojas-Tapias et al., 2022)	Primarily studied in the context of active inflammation (colitis); the threshold of inflammation and nitrite required to trigger this metabolic shift in a healthy or sub-clinically inflamed human gut is unclear.
*Prevotella intermedia* (Strain ATCC 25611)	Severe Bacteremic Pneumococcal Pneumonia	Respiratory tract	Adheres to respiratory epithelial cells (e.g., A549) via surface adhesins; uniquely upregulates PAFR transcription to facilitate *S. pneumoniae* invasion, leading to systemic bacteremia and cytokine storms (Nagaoka et al., 2014; Wu et al., 2021).	Active Pathogenicity	Confirmed (resident oral pathobiont translocated via microaspiration)	Viability & State: Culture-proven (viable respiratory co-colonization) Mechanistic Depth: Receptor-ligand binding / *In vitro* assay (upregulation of host epithelial PAFR expression) Interventional Validation: Murine intervention (*in vivo* co-infection models demonstrating increased lung/blood bacterial loads & mortality; Nagaoka et al., 2014; Wu et al., 2021) Human Evidence: Cross-sectional (airway aspiration & microbial co-occurrence profiling)	Most mechanistic steps of PAFR upregulation are characterized in murine systems; clinical verification of this synergism in human pneumococcal cohorts is limited.
*Fusobacterium nucleatum*	Colorectal Cancer (CRC) Enrichment	Gastrointestinal tract (Gut tumor microenvironment)	Fap2 adhesin binds to Gal-GalNAc on tumor surfaces (Abed et al., 2016). FadA binds E-cadherin to activate β -catenin (Rubinstein et al., 2013). Mechanistically demonstrated in murine colitis models: outer vesicles/bacteria provoke epithelial RIPK1-dependent necrosome assembly and lipid-peroxidation-driven ferroptosis (Zhang et al., 2025) and RIPK1-mediated death (Liu et al., 2021).	Active Pathogenicity	Confirmed (paired oral-colorectal metagenomic tracking)	Viability & State: Active metabolic adaptation (tumor niche survival & metabolic rewiring) Mechanistic Depth: Isogenic mutant (KO/rescue) ( ΔfadA,Δfap2 knockouts) & Receptor-ligand binding (FadA-E-cadherin, Fap2-Gal-GalNAc) Interventional Validation: Murine intervention (mouse xenograft models, $\mathrm{Ap}c^{\min/+}$ mice & metronidazole response) Human Evidence: Cross-sectional / Prospective cohort (human CRC cohort metagenomics & tumor biopsies; Fardini et al., 2011; Abed et al., 2016; Bullman et al., 2017; Liu et al., 2021; Zhang et al., 2025)	The contribution of oral translocated strains versus endogenous gut strains during the very early stages of CRC initiation is still being dissected.
	Adverse Pregnancy Outcomes & Preterm Birth	Placenta	Fap2 binds Gal-GalNAc on placental cells (Coppenhagen-Glazer et al., 2015). FadA adheres to endothelial cells, disrupting VE-cadherin to increase vascular permeability (Fardini et al., 2011).	Active Pathogenicity	Confirmed (paired maternal subgingival & placental strain concordance)	Viability & State: Culture-proven (viable bacterial isolation from placenta/amniotic fluid) Mechanistic Depth: Isogenic mutant (KO/rescue) ( Δfap2 and ΔfadA mutants) & Receptor-ligand binding (VE-cadherin disruption) Interventional Validation: Murine intervention (pregnant mouse transmission models causing fetal loss) Human Evidence: Cross-sectional (human placental PCR + clinical cohort association; Fardini et al., 2010; Coppenhagen-Glazer et al., 2015; Xu and Han, 2022; Kotzur et al., 2024)	Human placental models are ethically limited; *in vivo* studies rely on pregnant rodent models which differ in placental architecture.
*Porphyromonas gingivalis* (Strains W83 & 381)	Endothelial Dysfunction & Atherogenesis Acceleration	Arterial wall / Vascular endothelium	Invades human coronary artery endothelial cells (HCAECs) via endocytosis, persisting in autophagosomes/endosomes; LPS and gingipains stimulate TLR-NF- κ B-dependent endothelial-mesenchymal transition (EndMT); upregulates adhesion molecules (MCP-1, ICAM-1, VCAM-1), provokes iNOS/eNOS imbalance, oxidative stress, and caspase-8/9-mediated apoptosis; minor fimbriae (Mfa1) engage dendritic cell CD209 (DC-SIGN) to activate Akt/mTOR signaling and suppress autophagic clearance (Rodrigues et al., 2012; Meghil et al., 2019; Meghil and Cutler, 2020; Xie et al., 2020; Zhang et al., 2021).	Active Pathogenicity	Confirmed (Bacteremic entry following periodontal trauma or ulceration; subgingival plaque shedding)	Viability & State: Intracellular survival proven in cultured endothelial autophagosomes; 16S/PCR DNA detection in human atheromas. Mechanistic Depth: In vitro receptor-ligand binding (Mfa1–CD209), caspase-8/9 apoptosis assays, and EndMT cellular profiling. Interventional Validation: Murine intervention (*ApoE−/−* atherosclerosis mouse models with oral/intravenous bacterial challenge accelerating plaque maturation). Human Evidence: Systematic reviews and meta-analyses showing significant correlation between elevated serum anti-*P. gingivalis* IgG titers and coronary heart disease (CHD) risk (Rodrigues et al., 2012; Meghil et al., 2019; Meghil and Cutler, 2020; Xie et al., 2020; Zhang et al., 2021).	Preclinical causality is confirmed in *ApoE*−/−mice and cell lines; in human cohorts, evidence relies on antibody titers and plaque DNA detection, without direct proof that *Pg* independently initiates human atheroma rupture.
*Tannerella forsythia*	Atherosclerosis Progression	Vascular wall / Carotid plaque	Adheres via surface glycoproteins (SiaH, NanH); BspA protein triggers TLR2/MyD88 signaling to induce foam cell formation in macrophages (THP-1) and promote plaque maturation (Sharma, 2010; Lee et al., 2014).	Active Pathogenicity	Confirmed (core “red complex” periodontal pathogen)	Viability & State: DNA detection only in human atheroma (metagenomics/PCR); culture-based challenge in ApoE^−/−^ mouse models Mechanistic Depth: Receptor-ligand binding (BspA-TLR2 binding) & *In vitro* assay (macrophage THP-1 foam cell formation & methylglyoxal stress) Interventional Validation: Murine intervention (*in vivo* ${\text{ApoE}}^{-/-}$ atherosclerotic mouse models) Human Evidence: Cross-sectional (16S rRNA / metagenomic detection of bacterial DNA in carotid plaques; Rao et al., 2023)	Plaque colonization is largely validated in animal acceleration models; direct causal contribution to clinical plaque rupture in humans remains correlative.

This table applies a multidimensional evidentiary matrix to evaluate oral bacterial ectopic colonization and systemic disease associations. Ectopic Colonization differentiates Passive Detection / Associative (DNA detection or transient transit), Stable Colonization (viable persistence or metabolic adaptation), Active Pathogenicity (experimentally demonstrated tissue invasion, receptor engagement, or oncometabolite production in model systems), and Active Host Modulation (preclinical protective/probiotic immune regulation). Evidence of Oral Origin distinguishes Confirmed, Probable (shared lineages or anatomical contiguity), Associative (general translocation routes), and Primary Extra-oral (indigenous gut/urogenital taxa). The Evidence Basis explicitly separates preclinical experimental systems (in vitro, gnotobiotic/murine models) from human clinical cohorts to prevent overstating experimental causality as established human pathogenesis. Additional evidence-based evaluations of other oral bacterial ectopic colonizations are summarized in Supplementary File 1.

### 3.1 Streptococcus

The genus *Streptococcus* is a large group of common Gram-positive cocci, which are present in humans and animals, colonizing sites such as the nasopharynx and gastrointestinal tract, with most of them being the normal microbiota of the human body. *Streptococcus* is a main colonizer of the oral cavity, ubiquitously present as a key component of symbiotic oral biofilm microbiota. Its interaction with the host is paradoxical and strictly species-specific. On the one hand, commensal pioneer species—predominantly belonging to the mitis group (*Streptococcus sanguinis*, *Streptococcus gordonii*, *Streptococcus oralis*) and salivarius group (*Streptococcus salivarius*)—act as gatekeepers of homeostasis, essential for maintaining oral health—they shape the oral microbiota, modulate the immune system to permit normal bacterial survival, and combat pathogenic species (Bloch et al., 2024). Conversely, certain species in the oral cavity, such as *Streptococcus mutans* and *Streptococcus sobrinus*, are key components of the polymicrobial biofilm associated with dental caries (Leiva-Sabadini et al., 2024). While *in vitro* research indicates that these species produce acids capable of demineralizing tooth enamel, a clinical meta-analysis suggests that their abundance is associated with a higher risk of early childhood caries rather than establishing direct clinical causation (Manchanda et al., 2023). In periodontal disease, mechanistic studies suggest that oral streptococci serve as initial biofilm colonizers in the gingival pockets, facilitating the subsequent attachment of late-colonizing periodontal pathogens (Hajishengallis et al., 2011). Importantly, streptococcal species that primarily inhabit extra-oral niches—such as the intestinal and ruminal commensal *Streptococcus gallolyticus* (formerly *S. bovis* biotype I)—must be distinguished from true oral-derived taxa, as evidence supporting their oral-to-gut transmission remains uncertain.

Beyond the oral cavity, oral *Streptococcus* species are frequently detected in extra-oral sites via hematogenous dissemination. However, when evaluating their systemic pathogenicity, it is critical to distinguish between transient passive translocation (mere taxonomic detection) and stable ectopic colonization with proven functional impact on the host. While some systemic conditions are linked to these bacteria based on cross-sectional human studies (reflecting correlation), others are supported by rigorous *in vivo* animal models and multi-omics data demonstrating causal pathogenicity within defined experimental domains.

The most well-established example of functionally pathogenic ectopic colonization is infective endocarditis (IE). In IE, oral streptococci do not merely passively translocate; they actively adhere to heart valves and form persistent biofilms (Munro and Macrina, 1994). Recent transcriptomic and comparative genomic studies have elucidated these mechanisms, revealing that oral commensals, such as *Streptococcus sanguinis* and *Streptococcus gordonii*, undergo extensive transcriptional reprogramming when transitioning to the bloodstream (Baker et al., 2019). *In vitro* assays and *in vivo* experimental models of infective endocarditis have demonstrated that this transition includes the upregulation of genes governing oxidative stress responses and metal-ion-dependent adhesion via Type IV pili (specifically PilB), which functionally promote platelet binding and vegetation development in these experimental systems (Martini et al., 2021; Raynaud et al., 2021). Epidemiologically, a retrospective study of 569 clinical IE cases detected *Streptococcus oralis* in 104 cases (18.5%), indicating that *S. oralis* was associated with a substantial proportion of IE cases, which remained at a relatively high level in the statistical data from the periods 1987–1999 and 2000–2023 (Anguita Gámez et al., 2024).

Furthermore, recent metagenomic and metabolomic studies have provided compelling functional evidence for the stable ectopic colonization of oral streptococci in the gastrointestinal tract. For instance, multi-omics analyses have revealed that *Streptococcus anginosus* (a member of the *S. anginosus* group, SAG), a common oral pathobiont, is significantly enriched in patients with gastric cancer (GC) and colorectal cancer. Notably, a study by Fu et al. demonstrated that, in both conventional and germ-free mouse models, the oral pathobiont *S. anginosus* directly colonizes the gastric mucosa and drives chronic gastritis, atrophy, and dysplasia. Mechanistically, they showed that the *S. anginosus* surface protein TMPC directly binds to the Annexin A2 (ANXA2) receptor on gastric epithelial cells, which in turn triggers the activation of the mitogen-activated protein kinase (MAPK) signaling pathway to accelerate gastric tumorigenesis in these models (Fu et al., 2024). This provides mechanistic and experimental causal evidence in murine models demonstrating how ectopic *S. anginosus* can drive gastric pathology, though its definitive causative role in human gastric carcinogenesis remains to be further established in longitudinal clinical cohorts. Rather than being a passive bystander, metabolomic profiling and *in vivo* models demonstrate that *S. anginosus* promotes tumor cell proliferation by synthesizing specific oncogenic metabolites, such as methionine via the *metE* gene, providing functional evidence of its pathogenic impact in these experimental systems (Zhou et al., 2026).

In addition to cardiovascular and gastric niches, other oral streptococci display site-specific ectopic roles. For instance, *Streptococcus salivarius* has been experimentally linked to allergic rhinitis exacerbation via nasal Mucin5AC binding (Miao et al., 2023) and duodenal enteropathy in childhood stunting (Vonaesch et al., 2022), while *Streptococcus oralis* can synergize with *Candida albicans* to promote mucosal invasion (Diaz et al., 2012; Xu et al., 2016) or induce bladder oxidative stress in gnotobiotic mice (Nakamura et al., 2025).

### 3.2 Veillonella

The genus *Veillonella* consists of anaerobic Gram-negative cocci that act as core metabolic bridging commensals abundantly present in the human oral cavity. Their physiological characteristics include the active fermentation of organic acids (primarily lactic acid into weaker acetic and propionic acids) and the inability to utilize carbohydrates or amino acids as energy sources (Rogosa, 1964; Delwiche et al., 1985). *Veillonella* species (predominantly *Veillonella parvula*, *Veillonella dispar*, and *Veillonella rogosae*) are widely present in the oral cavity, pharynx, respiratory tract, and gastrointestinal tract. These bacteria play a significant role in oral microecology while also serving as conditional pathobionts in various oral and systemic microenvironments. In the oral cavity, *Veillonella* species display a paradoxical, niche-dependent role (Zhou et al., 2021). *In vitro* co-culture models show that *Veillonella parvula* can promote the formation of mature biofilms by *Streptococcus mutans*, enhancing its acid resistance, hydrogen peroxide detoxification, and overall biofilm virulence (Wei et al., 2024). In the altered microenvironment of Adult Severe Caries (ASC), experimental studies suggest that *V. parvula* can act as a co-pathogen, modulating the metabolic activity, spatial structure, and pathogenicity of *S. mutans* biofilms (Wei et al., 2024). Furthermore, in subgingival plaque, ecological models suggest that *Veillonella* species may act as potential heme suppliers supporting the growth of anaerobic periodontal pathogens, thereby facilitating biofilm maturation and contributing to periodontitis progression (Zhou et al., 2021).

Unlike the transient oral-to-gut translocation typically observed in healthy individuals, clinical association studies and animal experiments suggest that *Veillonella* can establish stable niches in the inflamed gut through specific metabolic adaptations. In a mechanistic study, *V. parvula* (strain DSM 2008) was shown to exploit the nitrate-rich environment created by intestinal inflammation. Specifically, using a murine model of colitis, Rojas-Tapias et al. demonstrated that *V. parvula* switches its metabolism from lactate fermentation to nitrate respiration; a mutant strain lacking the nitrate reductase gene (*narG* within the *narGHJI* operon) exhibited significantly reduced colonization capability in the mouse colon compared to the wild-type strain. This demonstrates in a murine colitis model that metabolic flexibility via nitrate respiration serves as a critical fitness factor facilitating its active colonization in the inflamed gut, rather than acting merely as an inert swallowed bystander (Rojas-Tapias et al., 2022).

In colorectal cancer (CRC), evidence is shifting from metagenomic association to functional validation. *V. parvula* has been shown to be enriched in CRC tissues. Furthermore, *in vitro* assays and murine models suggest that *V. parvula* can exert immunosuppressive effects, possibly by inhibiting the activation of B cells and macrophages to create an immune microenvironment favorable for intestinal tumorigenesis (Qian et al., 2023). This moves the understanding of *V. parvula* in CRC from a descriptive biomarker toward an active immune modulator in experimental systems. Similarly, in Pancreatic Ductal Adenocarcinoma (PDAC), a multinational metagenomic study of human cohorts identified oral *Veillonella* enrichment as a robust predictive signature across Japanese, Spanish, and German cohorts (Nagata et al., 2022). While clinical causation remains unproven, it is hypothesized that oral-derived strains might reach the pancreatic niche; however, whether local exposure induces dendritic cell activation (IL-6, TNF-α) to foster a pro-tumorigenic inflammatory microenvironment remains a hypothesis requiring direct experimental validation.

In contrast to the gut, evidence linking *Veillonella* to respiratory and neurological conditions remains largely associative. In the neonatal respiratory tract, high relative abundances of *Veillonella* (often co-occurring with *Prevotella* and *Streptococcus*) have been associated with an increased risk of asthma in children (Thorsen et al., 2023). However, it remains unclear whether *Veillonella* actively contributes to airway hyperresponsiveness or simply thrives in the altered mucosal environment of asthmatic patients. These instances represent classic opportunistic infections rather than specific pathogenesis. Other oral veillonellae similarly exhibit niche-specific associations without proven independent causality: *Veillonella dispar* correlates with pediatric caries status (Lee et al., 2025) and ulcerative colitis (Atarashi et al., 2017), *Veillonella rogosae* associates with pro-inflammatory dietary patterns and IBD relapse (da Rocha et al., 2023), while *Veillonella atypica* is enriched in fecal metagenomes of autoimmune myositis cohorts (Li et al., 2023).

### 3.3 Prevotella

*Prevotella* is a diverse genus of Gram-negative anaerobic bacteria, first described by Shah and Collins in 1990. This genus includes distinct oral and extra-oral lineages with specialized metabolic traits, such as moderate saccharolytic capability and bile-salt sensitivity in oral-adapted taxa (Tett et al., 2021). While *Prevotella* species colonize multiple mucosal interfaces and are linked to periodontitis, bacterial vaginosis, rheumatoid arthritis, and low-grade inflammation (Larsen, 2017), their functional properties and origins are strictly species-specific. Within the oral cavity, cross-sectional microbiome analyses show that *Prevotella intermedia* (a classic periodontal pathobiont) is significantly enriched in subgingival biofilms of patients with advanced periodontitis (Zhao et al., 2022; Ye et al., 2024). Conversely, in the supragingival and salivary niches, a prospective cohort study analyzing baseline saliva samples from 30 children found that caries-free children exhibited significantly higher relative abundances of *Prevotella nanceiensis* and *Prevotella melaninogenica* compared to those developing cavitated caries (Raksakmanut et al., 2023), suggesting that specific commensal oral *Prevotella* species may contribute to local microecological stability and caries resistance.

Outside the oral cavity, *Prevotella* species are detected across respiratory, gastrointestinal, and vascular compartments, displaying functional roles ranging from opportunistic synergy to protective immunomodulation (Figure 1). In the respiratory tract, oral *Prevotella* translocate via microaspiration, where episodic exposure can prime mucosal immunity (Wu et al., 2021). However, in acute polymicrobial infection, an intratracheal co-infection murine model provided experimental evidence of pathogenic synergy: mice co-inoculated with *Streptococcus pneumoniae* and *Prevotella intermedia* (ATCC 25611) exhibited significantly lower survival rates, elevated pulmonary and systemic bacterial burdens, and aggravated cytokine storms in bronchoalveolar lavage fluid (Nagaoka et al., 2014). Mechanistically, *P. intermedia* adheres to respiratory epithelial cells via surface adhesins, forms biofilms, and secretes proteases and cysteine endopeptidases that impair host defenses (Könönen et al., 2022). Notably, in human alveolar epithelial A549 cells, *P. intermedia* mechanistically upregulates host platelet-activating factor receptor (PAFR) transcription, directly facilitating pneumococcal adherence and transmigration—a synergistic virulence mechanism not induced by *Porphyromonas gingivalis* or *Fusobacterium nucleatum*.

**Figure 1:**
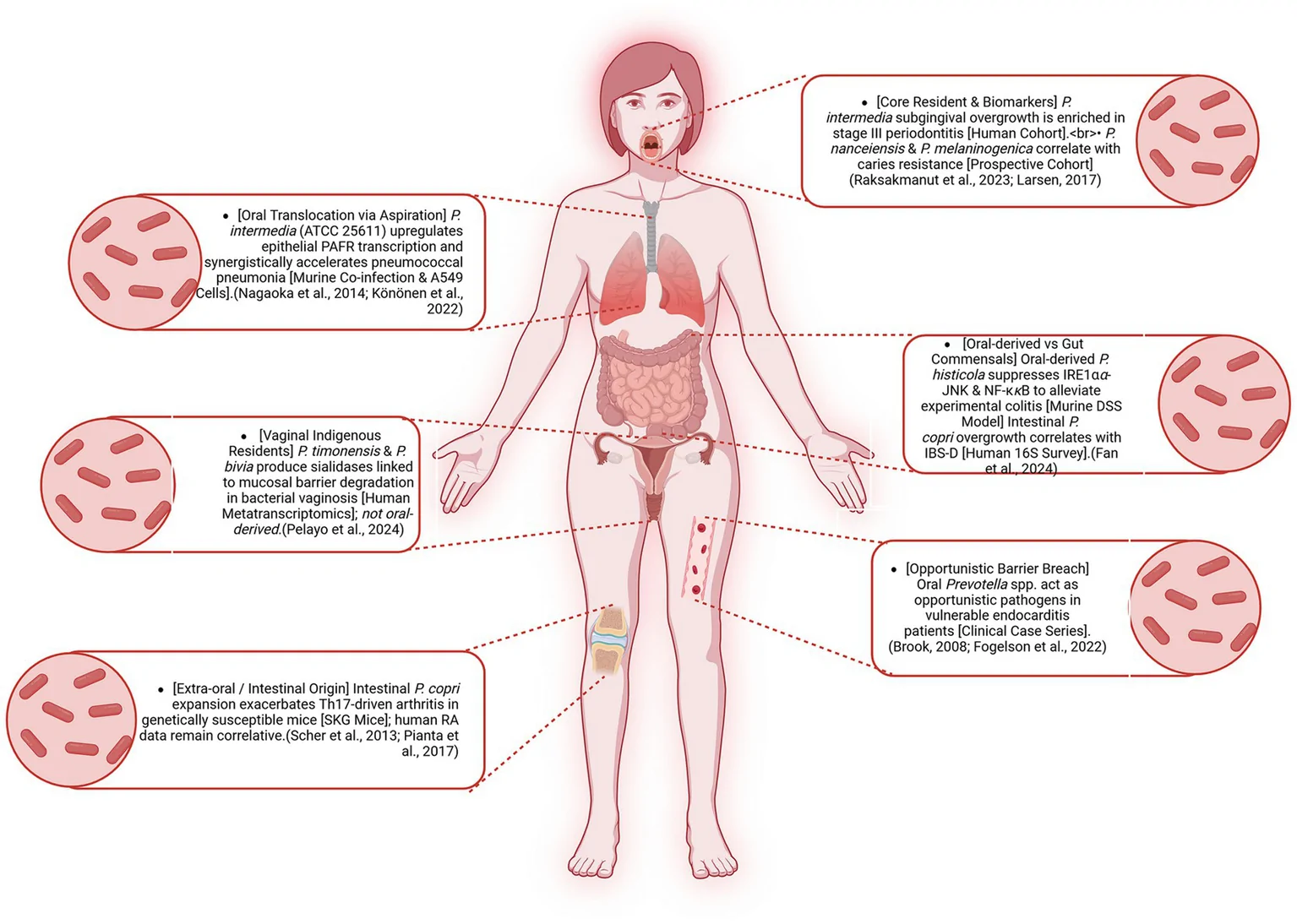
Systemic distribution, ecological niches, and disease associations of *Prevotella* species across experimental and clinical domains. Schematic overview illustrating the site-specific functional divergence and origin attribution of representative *Prevotella* lineages across human organ systems. Oral Cavity: *Prevotella intermedia* subgingival overgrowth is clinically enriched in stage III periodontitis cohorts, whereas commensal *Prevotella nanceiensis* and *Prevotella melaninogenica* in saliva correlate with caries resistance in prospective pediatric cohorts. Respiratory Tract upregulates host epithelial PAFR transcription and secretes cysteine endopeptidases, experimentally accelerating *Streptococcus pneumoniae* adherence, transmigration, and bacteremic pneumonia in murine co-infection models. Gastrointestinal Tract: Oral-derived *Prevotella histicola* suppresses mucosal IRE1 α -JNK endoplasmic reticulum stress and NF- κ B signaling, demonstrating protective anti-inflammatory efficacy in murine DSS-colitis models; in contrast, overgrowth of the indigenous colonic *Prevotella copri* complex correlates with diarrhea-predominant IBS in human 16S rRNA surveys. Female Reproductive Tract: Indigenous vaginal species *Prevotella timonensis* and *Prevotella bivia* abundantly express sialidases linked to cervical mucosal barrier breakdown in bacterial vaginosis and adverse pregnancy outcomes (*not oral-derived*). Joints: Intestinal expansion of *P. copri* exacerbates Th17-driven arthritis in genetically susceptible SKG mice, though human rheumatoid arthritis (RA) data remain correlative. Vascular System: Oral *Prevotella* spp. act as opportunistic bloodstream isolates in vulnerable patients with infective endocarditis. Created in BioRender.com. ATCC, American Type Culture Collection; BDM, brittle diabetes mellitus; BV, bacterial vaginosis; DSS, dextran sulfate sodium; IBD, inflammatory bowel disease; IBS-D, diarrhea-predominant irritable bowel syndrome; IRE1 α , inositol-requiring enzyme 1 α ; JNK, c-Jun N-terminal kinase; NF- κ B, nuclear factor kappa B; PAFR, platelet-activating factor receptor; RA, rheumatoid arthritis; Th17, T-helper 17 cell.

In the gastrointestinal tract, species of *Prevotella* display marked lineage-specific functional duality, requiring a strict distinction between oral translocators and extra-oral residents. The *Prevotella copri* complex (comprising multiple gut-resident clades) represents native colonic flora that ferment complex dietary fibers into short-chain fatty acids (SCFAs), but its overgrowth has also been associated with diarrhea-predominant IBS in fecal 16S rRNA surveys (Wu et al., 2019), and Th17-driven inflammation in susceptible arthritis models (Larsen, 2017; Tett et al., 2021). In contrast, true oral translocators like *P. intermedia* have been isolated from duodenal aspirates of stunted children; paired oral-gut metagenomics in the NEJM clinical cohort showed that these duodenal strains correlate with enteropathy and disrupt enterocyte barrier function (Chen et al., 2020). Conversely, *Prevotella histicola* (an oral mucosal commensal) demonstrates experimental protective efficacy: in a murine DSS-induced colitis model, orally administered *P. histicola* was mechanistically shown to ameliorate intestinal inflammation and maintain barrier integrity by suppressing the mucosal IRE1α-JNK endoplasmic reticulum stress and NF- κ B signaling pathways (Fan et al., 2024).

In the female reproductive tract, a metatranscriptomic analysis of vaginal mucosal samples by Pelayo et al. (2024) revealed that sialidase-encoding genes from *Prevotella timonensis* and *Prevotella bivia* are abundantly expressed during vaginal dysbiosis, correlating with compromised cervical mucus and adverse obstetric outcomes; however, these species represent resident vaginal taxa rather than oral-to-genital translocators. In the vascular compartment, resident oral *Prevotella* can occasionally enter the bloodstream following dental procedures or severe periodontal ulcerations; retrospective clinical isolate series indicate that vulnerable patients with pre-existing valvular defects or chronic immunosuppression have an increased clinical risk of developing subacute *Prevotella* infective endocarditis (Brook, 2008).

### 3.4 Lactobacillus

The genus *Lactobacillus* comprises Gram-positive, non-spore-forming bacilli. According to a literature review, these bacteria constitute a component of the human microbiota, with the oral cavity and gastrointestinal tract serving as typical anatomical colonization sites (Shah et al., 2024). In oral health, lactobacilli are widely explored for their antimicrobial and antagonistic properties. *In vitro* assays demonstrate that specific oral isolates, such as *Lacticaseibacillus rhamnosus* and *Lacticaseibacillus paracasei*, exhibit strong antagonistic activity against key periodontal pathobionts (Sookkhee et al., 2001). In periodontal clinical trials and animal models, topical administration of probiotic lactobacilli improves clinical indices, such as probing pocket depth, bleeding on probing, and experimental bone loss (Nguyen et al., 2021). Regarding dental caries, while specific strains antagonize *Streptococcus mutans* by suppressing *gtfB/C* expression *in vitro* (Strahinic et al., 2007; Wasfi et al., 2018), their high acidogenicity and aciduricity allow them to thrive as secondary colonizers in established dentinal carious lesions, exacerbating the low-pH microenvironment (Homayouni Rad et al., 2023). Furthermore, multi-species biofilm models demonstrate that adhesive interactions between *Lactobacillus* species, *Actinomyces*, and *S. mutans* can enhance structural biofilm biomass, increasing the risk of local cariogenic dysbiosis (Filoche et al., 2004; Wen et al., 2017).

Systemically, oral-associated lactobacilli present a distinct ecological paradigm that contrasts with invasive oral pathobionts. Unlike taxa equipped with specialized adhesins for active tissue breach, extra-oral detection of lactobacilli predominantly reflects two non-invasive pathways: passive gastrointestinal transit via swallowing and transient opportunistic bacteremia following mucosal disruption. In the gastrointestinal tract, swallowed oral lactobacilli constantly transit through the digestive tract; however, longitudinal multi-omics and metagenomic studies indicate that the majority fail to establish stable, persistent colonies in the distal gut of healthy immunocompetent hosts, largely acting as transient metabolic bystanders rather than permanent ectopic colonizers. Importantly, distinction must be made between resident oral strains and orally administered probiotic formulations, which display transient pharmacokinetics rather than stable colonization. Conversely, in the vascular system, severe periodontal ulceration, extensive dental extractions, or mucosal barrier breakdown can facilitate the hematogenous dissemination of resident oral lactobacilli (such as *Lacticaseibacillus rhamnosus*, *L. paracasei*, and *L. acidophilus*). Although lactobacilli exhibit low intrinsic virulence, clinical reviews and isolate-sequencing case series demonstrate that in vulnerable hosts—particularly those with preexisting valvular abnormalities, prosthetic devices, or profound immunosuppression—bloodstream-translocated oral strains can adhere to damaged endocardium, occasionally establishing persistent vegetative biofilms that lead to infective endocarditis (Kullar et al., 2023; Ioannou et al., 2024; Shah et al., 2024). Evaluating *Lactobacillus* within this evidentiary hierarchy underscores the crucial conceptual boundary between transient passive transit and functionally active, invasive ectopic pathogenicity.

### 3.5 Helicobacter pylori

*Helicobacter pylori* is a microaerophilic, spiral-shaped Gram-negative bacterium that colonizes the human gastric mucosa, affecting approximately 40 to 44% of the global population (Chen Y. C. et al., 2024). Although *H. pylori* is primarily an evolved gastric mucosal pathogen, its presence in the oral cavity—specifically within supragingival plaque, subgingival pockets, and saliva—has been widely documented via PCR and culture assays, though its status as a permanent resident versus a transiently regurgitated extragastric passenger remains a subject of ongoing debate (Yc et al., 2022). Observational reviews indicate that the oral presence of *H. pylori* is positively correlated with periodontitis severity (Maurotto et al., 2024). Supporting this association in a large clinical cohort, cross-sectional logistic regression of periodontal examinations and serology from the Third National Health and Nutrition Examination Survey (NHANES III) revealed that moderate-to-severe periodontitis was significantly associated with increased odds of *H. pylori* seropositivity compared to periodontal health (Sung et al., 2022). In the oral microenvironment, observational studies suggest that *H. pylori* may contribute to halitosis through bacterial urease-mediated urea hydrolysis into ammonia, while clinical correlations link its presence to the aggravation of oral lichen planus (OLP) and recurrent aphthous stomatitis, likely by inciting local pro-inflammatory cytokine release and mucosal microecological dysbiosis (Fan et al., 2025).

In the context of the oral-gut axis, *H. pylori* represents a unique model of bidirectional mucosal continuity and extragastric persistence, rather than a classical oral-derived colonizer originating *de novo* from the mouth. Although the human gastric mucosa serves as its primary evolutionary and ecological niche, persistent detection of *H. pylori* DNA and viable forms in supragingival plaque, subgingival biofilms, and saliva indicates that the oral microenvironment may act as an extragastric reservoir (Yc et al., 2022; Cuba et al., 2025; Fan et al., 2025). The central translational significance of oral *H. pylori* lies in its proposed role as an endogenous source for post-treatment gastric reinfection. Because systemic triple or quadruple antibiotic therapies exhibit limited penetration into mature, extracellular matrix-rich oral biofilms, viable *H. pylori* surviving in dental plaque can be continuously swallowed with saliva, potentially reseeding the cleared gastric mucosa and contributing to eradication failure or recurrent peptic ulcer disease. Clinical support for this bidirectional transmission model is provided by randomized controlled trials and systematic reviews: adjunctive nonsurgical periodontal therapy (scaling and root planing) combined with systemic eradication regimens is significantly associated with enhanced gastric *H. pylori* eradication rates and reduced long-term recurrence compared to systemic antibiotics alone (Maurotto et al., 2024; Cuba et al., 2025). Consequently, *H. pylori* exemplifies a primary extra-oral pathogen whose clinical management and recurrent pathology are intimately coupled to the barrier integrity and microecological dynamics of the oral cavity.

### 3.6 Fusobacterium nucleatum

As a Gram-negative anaerobic bacillus, the slender physical shape of *Fusobacterium nucleatum* enables its structural co-aggregation with various microbial cells within oral polymicrobial biofilms, such as dental plaque (Brennan and Garrett, 2019). In human clinical association studies, elevated oral *F. nucleatum* abundance is strongly associated with active periodontal and endodontic infections, including periodontitis, pulpitis, and periapical lesions (Nelson-Filho et al., 2018; Virkkunen et al., 2025). Mechanistic models have delineated these local pathological roles: *in vitro* cellular and murine models demonstrate that *F. nucleatum* triggers periapical tissue inflammation through Z-DNA binding protein 1 (ZBP1)-dependent pro-inflammatory cell death pathways (Liu et al., 2022). Furthermore, in a murine model of periodontitis, purified outer membrane vesicles (OMVs) secreted by *F. nucleatum* were shown to exacerbate alveolar bone loss and mucosal inflammation, displaying destructive effects comparable to the intact bacterium (Zhang et al., 2024b). Additionally, in an experimental rat model, *F. nucleatum*-induced periodontitis was found to exacerbate renal interstitial fibrosis via the TGF-β/SMAD2/3 and β-catenin signaling pathways (Chen P. et al., 2024). Similarly, *F. nucleatum* was experimentally shown to accelerate pulpitis progression in rats through a STING-dependent pathway (Zhou et al., 2024).

Although *F. nucleatum* is primarily an oral commensal, human association studies increasingly recognize it as an opportunistic pathobiont whose passive translocation or stable ectopic colonization is associated with various extra-oral conditions. Crucially, recent high-resolution genomic analyses demonstrate that systemic ectopic colonization is governed by strict subspecies- and clade-level genetic specializations. Metagenomic profiling of oral samples indicates that *Fusobacterium nucleatum* subsp. *polymorphum* (Fnp) dominates the healthy gingival margin, enriched in fluoride exporters and homeostatic biosynthetic gene clusters. In contrast, a landmark pangenomic and clinical metagenomic study by Zepeda-Rivera et al. revealed that *Fusobacterium nucleatum* subsp. *animalis* (Fna) comprises two genetically distinct clades: the Fna C1 clade remains predominantly confined to the oral cavity, whereas the Fna C2 clade is selectively enriched within the human colorectal cancer (CRC) tumor microenvironment. Genomic analyses demonstrated that the CRC-specialized Fna C2 clade has acquired defined metabolic adaptations—including enhanced gastric acid survival machinery and ethanolamine utilization operons—which facilitate stable, active ectopic colonization in the lower gastrointestinal tract rather than passive luminal transit (Zepeda-Rivera et al., 2024).

In the gastrointestinal tract, a high abundance of *F. nucleatum* in fecal or mucosal samples has been robustly associated with CRC and inflammatory bowel disease (IBD) in human cross-sectional cohorts (Fan et al., 2023). *In vitro* assays and mouse xenograft models demonstrate that *F. nucleatum* actively adheres to and invades transformed epithelial cells via specialized surface adhesins: FadA binds host E-cadherin to stimulate β -catenin oncogenic signaling (Rubinstein et al., 2013), while Fap2 selectively targets overexpressed Gal-GalNAc disaccharides on colorectal adenocarcinomas (Abed et al., 2016), forming persistent intratumoral biofilms and inducing pro-inflammatory cytokines (IL-6, IL-8) via host NF- κ B activation (Zhang et al., 2024a). Furthermore, integrated transcriptomic and metabolomic analyses show that *F. nucleatum*-derived vesicles remodel host central carbon metabolism to drive tumor cell proliferation *in vitro* (Wu et al., 2023). In tumor immunomodulation, clinical cohorts correlate intratumoral *F. nucleatum* enrichment with poor anti-PD-1 immunotherapy responses in metastatic CRC. Experimentally reconciling this phenomenon, Jiang et al. demonstrated in CT26 murine models that *F. nucleatum* ATCC 25586-derived succinic acid inhibits the host cGAS-interferon- β pathway, suppressing CD8 + T-cell infiltration and provoking immunotherapy resistance (Jiang et al., 2023). Conversely, Wang et al. reported in microsatellite stable (MSS) CRC murine models that specific butyrate-producing *F. nucleatum* isolates can augment anti-PD-1 therapeutic responses (Wang et al., 2024), While differences in bacterial clade genetic identity (such as Fna C2 vs. non-colonizing clades; Zepeda-Rivera et al., 2024), dominant metabolic profiles (succinate vs. butyrate), and tumor microsatellite stability represent biologically plausible hypotheses to explain these divergent outcomes, these variables have not yet been directly evaluated within a single standardized experimental system (Jiang et al., 2023; Wang et al., 2024). In experimental colitis models, *F. nucleatum* has been shown to exacerbate epithelial barrier breakdown via RIPK1-mediated necroptosis (Liu et al., 2021) and lipid-peroxidation-driven ferroptosis (Zhang et al., 2025).

In cardiovascular diseases, human cross-sectional studies have identified *F. nucleatum* as an enriched periodontitis-related pathogen in the oral cavity and atherosclerotic plaques (rather than the liver) of atherosclerosis patients. Moving beyond clinical correlation, interventional studies in mice demonstrate that *F. nucleatum*-induced periodontitis can mechanistically exacerbate atherosclerotic plaque formation and alter hepatic lipid metabolism by activating the PI3K/Akt/mTOR pathway (Zhou et al., 2023), providing a potential mechanistic link to MASLD.

In the context of pregnancy, human clinical association studies have found *F. nucleatum* enriched in various placental and fetal niches (Kotzur et al., 2024). Genomic analyses of strains detected in human amniotic fluid and placenta showed they match strains from maternal subgingival niches, indicating passive translocation from the oral cavity; this presence epidemiologically correlates with acute inflammation and adverse pregnancy outcomes (Xu and Han, 2022). Paired clonal strain analyses confirmed genomic identity between maternal subgingival isolates and placental strains, supporting hematogenous oral-to-placental translocation (Qian et al., 2023). In pregnant rodent models, intravenous inoculation of *F. nucleatum* demonstrated active fetal-placental tropism: bacterial FadA binds vascular endothelial cadherin (VE-cadherin) to increase endothelial permeability (Fardini et al., 2011), while Fap2 engages placental Gal-GalNAc to mediate localized colonization (Coppenhagen-Glazer et al., 2015), provoking severe placental inflammation and fetal demise in experimental systems (Fardini et al., 2010). While human evidence remains primarily associative due to ethical constraints on intervention during pregnancy, these animal models provide robust molecular mechanisms demonstrating the capacity of oral *F. nucleatum* to cross vascular-placental barriers during adverse pregnancy events.

### 3.7 Porphyromonas gingivalis

*Porphyromonas gingivalis* is a non-motile, asaccharolytic Gram-negative obligate anaerobic coccobacillus that derives energy exclusively from the fermentation of peptides and amino acids, relying on external sources of heme/iron and menadione (vitamin K) for growth. Within the subgingival pocket, *P. gingivalis* serves as the prototypical “keystone pathogen,” capable of orchestrating polymicrobial dysbiosis and destructive periodontitis even at low relative abundance by evading host immunity and remodeling the local symbiotic community (Reyes, 2021). In preclinical periodontitis models, selective targeting of *P. gingivalis* effectively suppresses inflammatory bone resorption and restores microbial homeostasis (Chen et al., 2023). In endodontic pathology, *in silico* molecular docking and computational simulations indicate that *P. gingivalis*-derived metabolites exhibit theoretical binding affinity for Wnt4, generating a hypothesis that it might disrupt pulpal regenerative cascades (Abulhamael et al., 2023). Furthermore, *in vitro* single-cell transcriptomic profiling of human dental pulp stem cells (hDPSCs) showed that exposure to *P. gingivalis* alters stem cell differentiation trajectories, providing a plausible cellular mechanism for impaired pulpal healing (Zhang et al., 2023). Additionally, *P. gingivalis* is frequently enriched in tumor tissues of oral squamous cell carcinoma (OSCC), where its chronic persistence correlates with cellular anti-apoptotic signaling and epithelial-mesenchymal transition (Fitzsimonds et al., 2020; Radaic et al., 2023).

Following bacteremia triggered by mastication or periodontal scaling, *P. gingivalis* or its outer membrane components can enter the bloodstream and disseminate to cardiovascular and distant niches. Clinical meta-analyses demonstrate that elevated serum IgG antibody titers against *P. gingivalis* correlate with increased coronary heart disease risk, reflecting systemic immunogenic exposure rather than direct atheromatous causality (Joshi et al., 2021; Li et al., 2021; Aleksijević et al., 2022). *In vitro* cellular and rodent models demonstrate that *P. gingivalis* actively invades human coronary artery endothelial cells (HCAECs) via endocytic pathways, persisting within autophagosomes and late endosomes. Notably, strain-specific virulence dictates intracellular persistence: the encapsulated strain W83 and fimbriated strain 381 exhibit distinct transcriptional reprogramming to evade phagolysosomal degradation, facilitating intracellular survival (Rodrigues et al., 2012). In experimental cellular and animal models, *Porphyromonas gingivalis* is suggested to stimulate endothelial cells to highly express adhesion molecules (e.g., MCP-1, ICAM-1, and VCAM-1), thereby enhancing monocyte migration and adhesion, which may contribute to the initiation and progression of atherosclerosis (Zhang et al., 2021). On the other hand, *in vitro* experiments indicate that this bacterium can induce oxidative stress in endothelial cells, which is associated with endothelial dysfunction (Zhang et al., 2021). Simultaneously, *Porphyromonas gingivalis* has been shown to inhibit endothelial cell proliferation, induce endothelial-mesenchymal transition (EndMT) in a TLR-NF-κB-dependent manner (Xie et al., 2020), and activate both the caspase-8 death receptor and caspase-9 mitochondrial-dependent apoptotic pathways (Zhang et al., 2021), leading to reduced cell viability and impaired vascular endothelial barrier function *in vitro*. Importantly, studies have also found that *Porphyromonas gingivalis* can bind to CD209 on the surface of dendritic cells (DCs) via its Mfa1 fimbriae (Meghil and Cutler, 2020). *In vitro* experiments further demonstrate that this interaction activates DCs and promotes inflammatory responses, while modulating the Akt/mTOR signaling pathway to alter autophagy and apoptosis, which is hypothesized as a mechanism for evading immune surveillance (Meghil et al., 2019; Figure 2).

**Figure 2:**
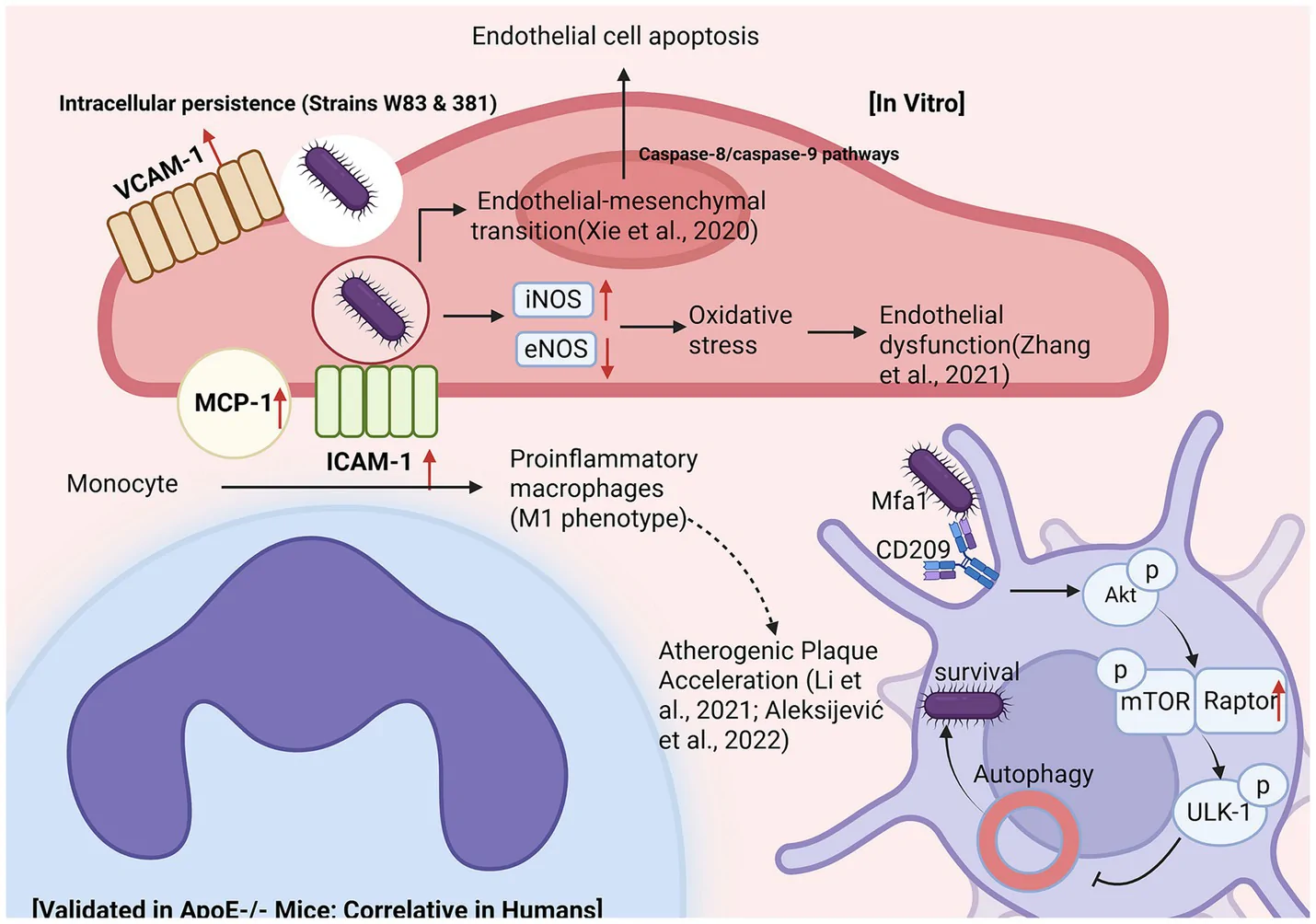
Experimental and *in vitro* mechanisms of *Porphyromonas gingivalis* in endothelial injury and immune evasion. Detailed molecular signaling pathways characterized in human endothelial cell cultures, dendritic cells, and preclinical rodent models. Vascular Endothelial Injury (*In Vitro* & *ApoE−/-*Models): Invading *P. gingivalis* (strains W83 and 381) establishes intracellular persistence within autophagosomes and vacuoles. Exposure to bacterial LPS and gingipains stimulates endothelial cells to upregulate adhesion molecules (MCP-1, ICAM-1, VCAM-1), promoting monocyte recruitment and pro-inflammatory M1 macrophage polarization. Concurrently, intracellular infection induces an iNOS/eNOS enzyme imbalance, triggering reactive oxygen species (ROS)-mediated oxidative stress, TLR-NF- κ B-dependent endothelial-mesenchymal transition (EndMT), and caspase-8/caspase-9-dependent apoptosis, compromising vascular barrier integrity. Dendritic Cell Immune Evasion (*In Vitro* Assays): Engagement of bacterial minor fimbriae (Mfa1) with dendritic cell CD209 (DC-SIGN) receptors triggers the intracellular Akt/mTOR/Raptor phosphorylation cascade, which in turn phosphorylates and inactivates ULK-1, suppressing autophagosome maturation and promoting intracellular bacterial survival. Evidentiary Bounding: Solid arrows denote experimentally validated molecular and cellular cascades; the dashed arrow indicates the hypothesized link from macrophage activation to clinical atherogenic plaque acceleration (*validated in ApoE*−/− *mice; correlative in human cohorts*). Created in BioRender.com. *ApoE*, apolipoprotein E; CD209, cluster of differentiation 209 (DC-SIGN); DC, dendritic cell; EndMT, endothelial-mesenchymal transition; eNOS, endothelial nitric oxide synthase; HCAEC, human coronary artery endothelial cell; ICAM-1, intercellular adhesion molecule-1; iNOS, inducible nitric oxide synthase; LPS, lipopolysaccharide; M1, pro-inflammatory type 1 macrophage; MCP-1, monocyte chemoattractant protein-1; Mfa1, minor fimbrial antigen 1; mTOR, mammalian target of rapamycin; NF- κ B, nuclear factor kappa B; Raptor, regulatory-associated protein of mTOR; TLR, Toll-like receptor; ULK-1, unc-51 like autophagy activating kinase 1; VCAM-1, vascular cell adhesion molecule-1.

Subsequently, in metabolic diseases, evidence from *in vivo* interventional murine models suggests that *Porphyromonas gingivalis* infection exacerbates Metabolic dysfunction-associated steatotic liver disease (MASLD) progression, with reviews highlighting its surface components (e.g., fimbriae, outer membrane protein A, and lipopolysaccharide) as major pathogenic factors (Hasegawa and Nagano, 2021; Lunar Silva and Cascales, 2021). In these animal models, these components are proposed to promote MASLD development through mechanisms including endotoxemia, inflammatory responses, oxidative stress, metabolic remodeling, M1 macrophage polarization, and gut microbiota disruption (Mei et al., 2020; Ahn et al., 2021; Hatasa et al., 2021). Providing multi-omics functional evidence, Wu et al. revealed in HFD mice that intracellular *P. gingivalis* upregulates bacterial branched-chain amino acid (BCAA) ABC transporter genes (*livH* and *livK*), increasing circulating host BCAA levels and aggravating steatohepatitis—a phenotype reversed by *livH/livK* gene silencing in experimental models (Wu et al., 2022). Along the oral-gut-brain axis, Ma et al. demonstrated in mice that *P. gingivalis* outer membrane vesicles translocate to the brain parenchyma via the trigeminal nerve and periodontal vasculature, activating microglia and inducing experimental cognitive decline (Ma et al., 2023). Clinically, multi-cohort metagenomic profiling by Clasen et al. identified oral-gut microbial virulence signatures that correlate with neuroinflammatory markers in Parkinson’s disease cohorts, though causal mechanisms in humans remain under investigation (Clasen et al., 2025). In obstetric models, oral or intravenous administration of *P. gingivalis* in pregnant mice induced placental colonization, shifted the placental Th1/Th2 cytokine ratio (upregulating IFN- γ and IL-2), and provoked gestational metabolic disturbances leading to fetal growth restriction (Yoshida et al., 2021). While human placental PCR surveys occasionally detect *P. gingivalis* DNA in preeclampsia biopsies (Barak et al., 2007), human clinical intervention trials show mixed efficacy in reducing adverse birth outcomes, underscoring that preclinical rodent models reflect experimental biological plausibility rather than established clinical causality.

### 3.8 Tannerella forsythia

*Tannerella forsythia* (referring to the bacterium) belongs to the Cytophaga-Bacteroides family and is an anaerobic Gram-negative bacterium. Initially described and named *Bacteroides forsythus* by Tanner et al. (1986), it was later reclassified as *Tannerella forsythia* by Sakamoto *et al.* based on 16S rRNA phylogenetic analysis (Sakamoto et al., 2002). Physiologically, *T. forsythia* exhibits a distinct metabolic auxotrophy, requiring exogenous *N*-acetylmuramic acid (MurNAc)—an essential peptidoglycan precursor typically scavenged from co-habiting biofilm partners (such as *P. gingivalis* or *F. nucleatum*)—for cell wall synthesis and propagation. Within the subgingival pocket, *T. forsythia* constitutes a classic member of Socransky’s pathogenic “red complex” (alongside *P. gingivalis* and *Treponema denticola*), strongly associated with advanced pocket depth and active alveolar bone loss in progressive periodontitis (Ardila and Bedoya-García, 2020).

Beyond the oral cavity, *T. forsythia* can translocate to extra-oral mucosal sites via aspiration or local spread. Clinically, metagenomic next-generation sequencing (mNGS) identified viable *T. forsythia* mono-infection in a human lung abscess, confirming its opportunistic pathogenic potential in the lower respiratory tract following microaspiration (Miao et al., 2024) suggesting its potential translocation to extrapulmonary niches such as the respiratory tract. Furthermore, although *T. forsythia* is primarily an oral anaerobe, experimental animal studies have shown that subcutaneous inoculation of this bacterium can induce skin abscesses in mice and rabbits, demonstrating its pathogenic potential outside the oral cavity under specific experimental conditions. *In vitro* mechanistic studies suggest that its pathogenic mechanisms primarily involve adhesion mediated by surface proteins and glycosidases, such as *siaH* and *nanH*, which were shown to degrade glycoproteins on host cell surfaces to expose additional binding sites and facilitate bacterial attachment (Schäffer and Andrukhov, 2024). Notably, *in vitro* assays demonstrated that *T. forsythia* grown in glucose-supplemented media produces methylglyoxal, a reactive dicarbonyl compound hypothesized to promote tissue damage through advanced glycation end-product formation and protein cross-linking (Sharma, 2010; Figure 3). In a rat catch-up fat model, topical dorsal tongue inoculation with *T. forsythia* experimentally promoted high-fat dietary intake and aggravated insulin resistance (Hu et al., 2018), suggesting a potential behavioral-metabolic feedback loop that warrants validation in clinical cohorts.

**Figure 3:**
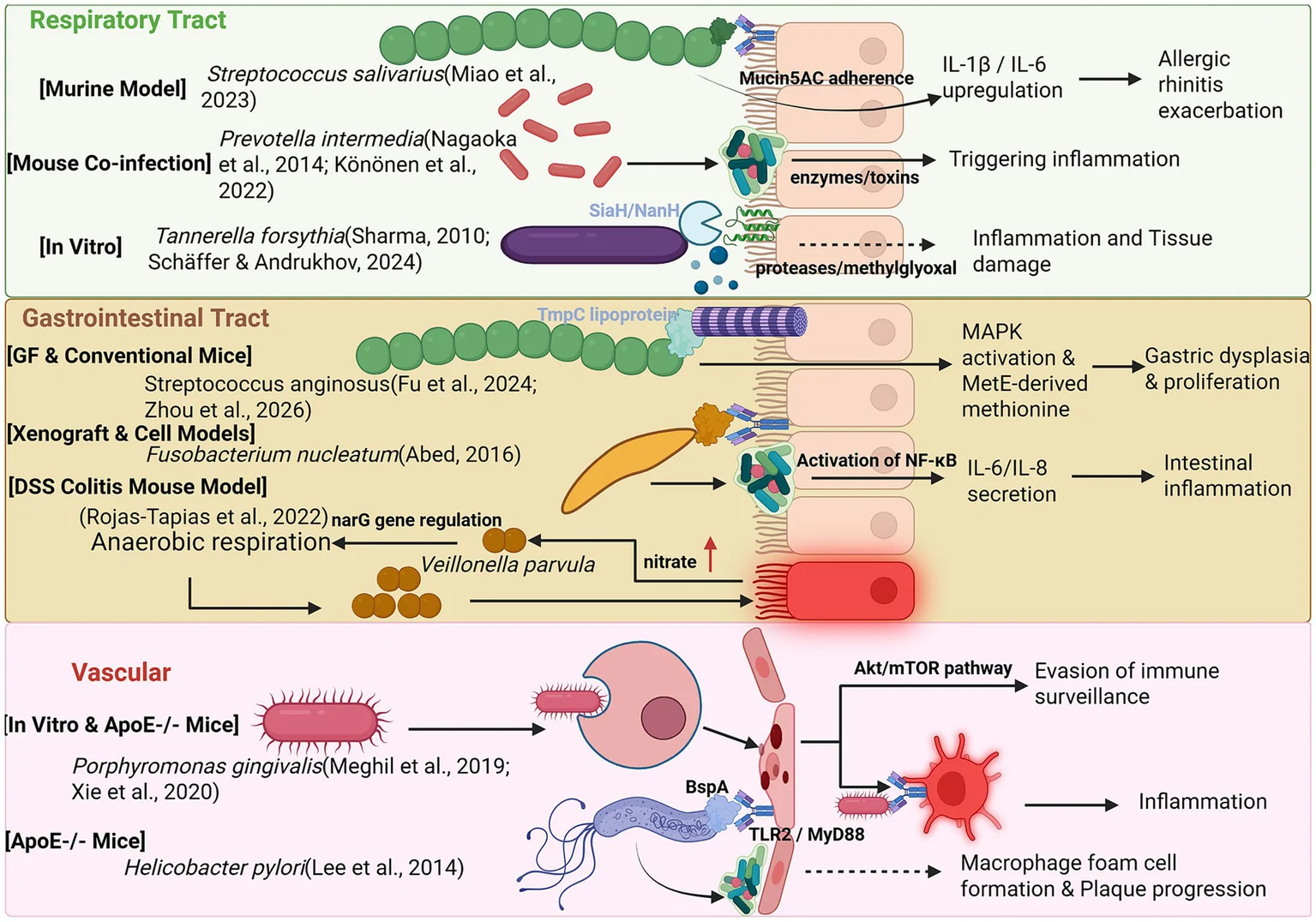
Tract-specific molecular strategies and experimental virulence mechanisms of ectopic oral taxa across mucosal and vascular compartments. Schematic overview of species-resolved virulence mechanisms validated in cellular and animal models across three major anatomical tracts. Respiratory Tract: *Streptococcus salivarius* engages host epithelial Mucin5AC to upregulate IL-1 β and IL-6, exacerbating allergic rhinitis [Murine Model]; *Prevotella intermedia* secretes proteases and toxins, triggering local inflammation and priming pneumococcal invasion [Mouse Co-infection]; *Tannerella forsythia* deploys SiaH/NanH sialidases to cleave protective mucosal glycans and releases methylglyoxal [*In Vitro*]. Gastrointestinal Tract: *Streptococcus anginosus* utilizes surface lipoprotein TmpC to bind epithelial ANXA2, activating MAPK signaling and synthesizing *metE*-derived methionine to drive gastric dysplasia and proliferation [GF & Conventional Mice]; *Fusobacterium nucleatum* (Fna C2 clade) engages epithelial E-cadherin via FadA to trigger host NF- κ B activation and IL-6/IL-8 secretion [Xenograft & Cell Models]; *Veillonella parvula* exploits inflammation-derived nitrate to upregulate the *narGHJI* operon (*narG*), switching from lactate fermentation to anaerobic nitrate respiration for stable colonization [DSS Colitis Mouse Model]. Vascular System: *Porphyromonas gingivalis* activates the intracellular Akt/mTOR pathway to evade immune surveillance and incites endothelial inflammation [*In Vitro* & ApoE−/− Mice]; *Tannerella forsythia* deploys surface leucine-rich repeat protein BspA to engage host TLR2/MyD88 signaling, driving macrophage foam cell formation and plaque progression [ApoE−/− Mice]. Evidentiary Bounding: Solid arrows represent experimentally confirmed pathways; dashed arrows denote putative tissue damage or plaque maturation cascades requiring further clinical validation. Created in BioRender.com. ANXA2, annexin A2; *ApoE*, apolipoprotein E; BspA, bacterial surface protein A; DSS, dextran sulfate sodium; GF, germ-free; IL-6/8, interleukin-6/8; MAPK, mitogen-activated protein kinase; Mfa1, minor fimbrial antigen 1; mTOR, mammalian target of rapamycin; *narG*, nitrate reductase alpha subunit; NF- κ B, nuclear factor kappa B; PAFR, platelet-activating factor receptor; SiaH/NanH, sialidases/neuraminidases; TLR2, Toll-like receptor 2; TmpC, transmembrane protein C.

In the cardiovascular system, 16S rRNA and metagenomic sequencing surveys have detected *T. forsythia* DNA within human carotid and coronary atherosclerotic plaques, reflecting transient bacteremic shedding from ulcerated periodontal pockets rather than persistent tissue colonization (Rao et al., 2023). Providing experimental mechanistic plausibility, interventional studies using *ApoE^−/−^* mice and human THP-1 macrophage cultures demonstrated that *T. forsythia* and its cell-surface leucine-rich repeat (LRR) protein BspA directly activate Toll-like receptor 2 (TLR2)/MyD88 signaling, driving lipid accumulation, foam cell formation, and accelerated atheromatous plaque maturation in these preclinical models (Lee et al., 2014). While these findings establish a clear molecular mechanism in cellular and rodent systems, the presence of *T. forsythia* in human atheromas remains correlative, and whether active periodontal eradication directly retards clinical plaque progression warrants prospective interventional trials.

### 3.9 Physiological commensalism and context-dependent symbiotic roles

Under physiological conditions, many of the aforementioned oral taxa are not merely passive residents or subclinical pathogens; they actively participate in maintaining host homeostasis, mucosal barrier integrity, and niche exclusion.

For instance, although *Veillonella* species (such as *V. parvula*) are frequently enriched in active carious lesions, their baseline physiological role is highly protective. *Veillonella* cannot utilize sugars for fermentation; instead, they rely on lactic acid—the strongest demineralizing acid produced by cariogenic streptococci—converting it into weaker propionic and acetic acids (Rogosa, 1964). This metabolic syntrophy acts as a physiological acid buffer, raising the local microenvironmental pH and reducing enamel demineralization under steady-state conditions. The apparent contradiction between its protective role as an “acid sink” in health and its enrichment in severe caries is a classic example of context-dependency. When dietary sucrose is excessive, the physical and metabolic co-aggregation between *S. mutans* and *V. parvula* accelerates biofilm biomass accumulation; *V. parvula* upregulates metabolic stress-tolerance and transport pathways to thrive within the highly acidic, sucrose-rich dysbiotic plaque, which in turn facilitates the survival of aciduric pathogens and worsens demineralization despite the bacteria’s weaker acid conversion (Wei et al., 2024).

Similarly, *Fusobacterium nucleatum*, while recognized as an opportunistic pathobiont in periodontitis and extra-oral tumors, serves a vital protective role in healthy oral mucosal tissue. Under steady-state conditions, non-invasive oral clades—predominantly *Fusobacterium nucleatum* subsp. *polymorphum*—deploy their cell wall-associated diacylated lipoprotein FAD-I to strongly induce human beta-defensin-2 (hBD-2) and hBD-3 in human oral epithelial cells via TLR-1/2 and TLR-2/6 signaling pathways (Bhattacharyya et al., 2016). This homeostatic induction of host antimicrobial peptides strengthens the epithelial barrier and protects oral tissues from invasion by sensitive periodontopathogens, such as *Porphyromonas gingivalis*. This protective barrier-building function is hypothesized to shift into pathogenic facilitation only when mucosal barriers are breached or when subgingival ecological dysbiosis permits hyper-aggregation with bridging partners.

Furthermore, pioneer oral colonizers like *Streptococcus salivarius* demonstrate robust symbiotic and protective functions under physiological conditions. Specific commensal *S. salivarius* strains (such as K12 and M18) produce bacteriocins and bacteriocin-like inhibitory substances (BLIS), including salivaricin A2, B, and G32 encoded by megaplasmids, which actively suppress virulent upper respiratory pathogens like *Streptococcus pyogenes* (Wescombe et al., 2006). Additionally, *in vitro* studies using epithelial cell co-cultures demonstrate that live *S. salivarius* (e.g., strain JIM8772) can downregulate host NF- κ B reporter activation and inhibit IL-8 secretion (Kaci et al., 2014), highlighting its potential role in maintaining mucosal immune tolerance. By recognizing these dual, context-dependent properties, we can avoid oversimplifying these microbes as absolute pathogens and instead view them as ecological gatekeepers whose pathogenic potential is unlocked only under specific physiological or environmental dysbiosis.

## 4 Conclusion

As an indispensable component of the human microbiota ecosystem, the oral microbiota plays a pivotal role in maintaining oral local homeostasis and overall health: through its own colonization, expansion, and the cross-organ interaction networks formed with other organs, it not only acts as a key regulatory factor in local oral diseases such as dental caries and periodontitis, but also participates in the pathophysiological processes of systemic diseases including colitis and Metabolic-Associated Fatty Liver Disease. Notably, the discovery of the oral-gut-liver axis has further provided a novel perspective for unraveling the pathogenesis of microbiota-mediated diseases, while also pointing to multi-dimensional directions for future research and application. Despite the growing body of literature linking oral microbiota to extra-oral conditions, current evidence presents several limitations. A significant portion of the data is compositional, derived from cross-sectional associations rather than longitudinal studies, making it difficult to establish definitive causality. Furthermore, many mechanistic claims rely heavily on animal models under artificial conditions, which may not fully replicate human host-microbiome interactions.

To overcome these limitations, future investigations must transition from cross-sectional, bulk compositional profiling to high-resolution functional frameworks. This will necessitate paired oral-extraoral strain-resolved sampling via deep shotgun metagenomics to precisely track clonal transmission and distinguish true ectopic colonization from mere taxonomic correlation. Furthermore, the routine integration of viability assays, such as propidium monoazide (PMA)-based metagenomics, will be crucial to differentiate intact, active translocated pathobionts from nonviable DNA fragments shedding into low-biomass distant tissues. Concurrently, the deployment of metatranscriptomics and metabolomics along the oral-extraoral axes can provide invaluable insights into *in situ* microbial transcriptional activity and functional metabolite dynamics (e.g., short-chain fatty acids, succinate, and bile acids) within colonized niches. Such efforts should be complemented by the design of dense longitudinal cohorts to map temporal colonization trajectories and validate disease-onset correlation.

On the clinical front, while targeting microbiota-organ interaction pathways holds immense promise, we must caution against premature clinical extrapolation. Technological innovations, such as microbiome diagnostic chips based on oral swabs combined with machine learning, must be rigorously trained to differentiate speculative candidates from reproducible, multi-cohort validated biomarkers. Ultimately, translating these findings into precision prevention and treatment protocols will require robust, randomized intervention studies to ascertain how oral hygiene improvements, specific antimicrobials, or pre/probiotics causally modulate systemic health. By refining our evidentiary standards, we can pave new, scientifically sound avenues for the accurate management of oral-systemic diseases.
