# Supplementary material for: Functional heterogeneity and health impacts of oral bacterial ectopic colonization
Source: Front Microbiol. 2026 Sep 25;17:1915341. doi: 10.3389/fmicb.2026.1915341 (PMC13650913; doi:10.3389/fmicb.2026.1915341)
Supplement: Supplementary file 1 [file Supplementary_file_1.DOCX]

**Supplementary Material S1**

**Literature Search Strategy, Platform-Specific Search Strings, and Evidence Evaluation Framework**

**1. Literature Retrieval Overview**

To ensure transparency and breadth across multi-organ axes, a concept-driven literature search was conducted across PubMed/MEDLINE, Embase, Web of Science Core Collection, and Google Scholar from inception to January 2026. The search was restricted to peer-reviewed English-language publications.

The search architecture combined three core thematic domains using Boolean AND logic:

**Domain 1 (Oral Taxa):** Descriptors of oral microecology and the eight focused bacterial taxa (*Streptococcus*, *Veillonella*, *Prevotella*, *Lactobacillus* [including reclassified taxa such as *Lacticaseibacillus* and *Lactiplantibacillus*], *Helicobacter pylori*, *Fusobacterium nucleatum*, *Porphyromonas gingivalis*, and *Tannerella forsythia*).

**Domain 2 (Translocation & Niche Dynamics):** Controlled terms and keywords for spatial dissemination, mucosal barrier breach, and metabolic adaptation (e.g., *"ectopic colonization"*, *"bacterial translocation"*, *"oral-gut-liver axis"*, *"metabolic adaptation"*).

**Domain 3 (Target Pathologies & Systemic Outcomes):** Subject headings and keywords covering cardiovascular, gastrointestinal, hepatic, respiratory, neurological, obstetric, and metabolic conditions.

**2. Platform-Specific Executable Search Strategies**

**2.1 PubMed / MEDLINE**

("Oral Microbiome"[tiab] OR "Oral Cavity"[tiab] OR "Dental Plaque"[Mesh] OR "Saliva"[Mesh] OR "Streptococcus"[Mesh] OR "Streptococcus"[tiab] OR "Veillonella"[Mesh] OR "Veillonella"[tiab] OR "Prevotella"[Mesh] OR "Prevotella"[tiab] OR "Lactobacillus"[Mesh] OR "Lactobacillus"[tiab] OR "Lacticaseibacillus"[tiab] OR "Lactiplantibacillus"[tiab] OR "Helicobacter pylori"[Mesh] OR "Helicobacter pylori"[tiab] OR "Fusobacterium nucleatum"[Mesh] OR "Fusobacterium nucleatum"[tiab] OR "Porphyromonas gingivalis"[Mesh] OR "Porphyromonas gingivalis"[tiab] OR "Tannerella forsythia"[Mesh] OR "Tannerella forsythia"[tiab])

AND

("Bacterial Translocation"[Mesh] OR "Bacterial Translocation"[tiab] OR "Ectopic colonization"[tiab] OR "Oral-gut axis"[tiab] OR "Oral-gut-liver axis"[tiab] OR "Oral-gut-brain axis"[tiab] OR "Hematogenous dissemination"[tiab] OR "Microaspiration"[tiab] OR "Metabolic adaptation"[tiab] OR "Anaerobic respiration"[tiab] OR "Virulence factors"[tiab])

AND

("Endocarditis, Bacterial"[Mesh] OR "Infective endocarditis"[tiab] OR "Atherosclerosis"[Mesh] OR "Atherosclerosis"[tiab] OR "Stroke"[Mesh] OR "Colorectal Neoplasms"[Mesh] OR "Colorectal cancer"[tiab] OR "Stomach Neoplasms"[Mesh] OR "Gastric cancer"[tiab] OR "Inflammatory Bowel Diseases"[Mesh] OR "Colitis"[Mesh] OR "Non-alcoholic Fatty Liver Disease"[Mesh] OR "NAFLD"[tiab] OR "MASLD"[tiab] OR "Pneumonia"[Mesh] OR "Rhinitis, Allergic"[Mesh] OR "Asthma"[Mesh] OR "Lung Abscess"[Mesh] OR "Alzheimer Disease"[Mesh] OR "Parkinson Disease"[Mesh] OR "Premature Birth"[Mesh] OR "Preterm birth"[tiab] OR "Failure to Thrive"[Mesh] OR "Childhood stunting"[tiab] OR "Insulin Resistance"[Mesh] OR "Rheumatoid Arthritis"[Mesh] OR "Myositis"[Mesh] OR "Bacteremia"[Mesh])

**2.2 Embase (Elsevier)**

('mouth flora'/exp OR 'saliva'/exp OR 'dental plaque'/exp OR 'streptococcus'/exp OR 'veillonella'/exp OR 'prevotella'/exp OR 'lactobacillus'/exp OR 'lacticaseibacillus':ti,ab,kw OR 'lactiplantibacillus':ti,ab,kw OR 'helicobacter pylori'/exp OR 'fusobacterium nucleatum'/exp OR 'porphyromonas gingivalis'/exp OR 'tannerella forsythia'/exp)

AND

('bacterial translocation'/exp OR 'ectopic colonization':ti,ab,kw OR 'oral gut axis':ti,ab,kw OR 'oral-gut-liver axis':ti,ab,kw OR 'bloodstream dissemination':ti,ab,kw OR 'microaspiration':ti,ab,kw OR 'metabolic adaptation':ti,ab,kw)

AND

('bacterial endocarditis'/exp OR 'atherosclerosis'/exp OR 'cerebrovascular accident'/exp OR 'colorectal tumor'/exp OR 'stomach cancer'/exp OR 'inflammatory bowel disease'/exp OR 'nonalcoholic fatty liver'/exp OR 'pneumonia'/exp OR 'allergic rhinitis'/exp OR 'asthma'/exp OR 'lung abscess'/exp OR 'alzheimer disease'/exp OR 'parkinson disease'/exp OR 'prematurity'/exp OR 'growth retardation'/exp OR 'insulin resistance'/exp OR 'rheumatoid arthritis'/exp OR 'bacteremia'/exp)

AND [english]/lim

**2.3 Web of Science Core Collection (Clarivate)**

TS=(("Oral microbiome" OR "Oral cavity" OR "Streptococcus" OR "Veillonella" OR "Prevotella" OR "Lactobacillus" OR "Lacticaseibacillus" OR "Lactiplantibacillus" OR "Helicobacter pylori" OR "Fusobacterium nucleatum" OR "Porphyromonas gingivalis" OR "Tannerella forsythia"))

AND

TS=(("Bacterial translocation" OR "Ectopic colonization" OR "Oral-gut axis" OR "Oral-gut-liver axis" OR "Oral-gut-brain axis" OR "Hematogenous dissemination" OR "Microaspiration" OR "Metabolic adaptation"))

AND

TS=(("Infective endocarditis" OR "Atherosclerosis" OR "Stroke" OR "Colorectal cancer" OR "Gastric cancer" OR "Inflammatory bowel disease" OR "Colitis" OR "MASLD" OR "Pneumonia" OR "Allergic rhinitis" OR "Asthma" OR "Alzheimer*" OR "Parkinson*" OR "Preterm birth" OR "Childhood stunting" OR "Insulin resistance" OR "Rheumatoid arthritis" OR "Bacteremia"))

**3. Study Selection & Appraisal Principles**

Because this narrative review aims at conceptual synthesis and mechanistic clarity rather than exhaustive epidemiological quantification, literature selection was conducted purposively.

**Prioritization Criteria:** We prioritized peer-reviewed original studies providing:

Paired oral–extraoral strain-level resolution (e.g., shotgun metagenomics or isolate sequencing);

Defined molecular mechanisms in *in vitro* or isogenic mutant ($\Delta gene$) models;

Causal interventional validation in animal systems (e.g., gnotobiotic, knockout, or disease models);

Well-characterized prospective human clinical cohort associations.

**Exclusion Criteria:** Localized oral studies without systemic/extra-oral evaluations, non-peer-reviewed preprints, conference abstracts lacking verifiable experimental data, and non-English publications.

**Screening Workflow:** Literature evaluation and data extraction were performed collaboratively by two authors (K.X. and M.J.), with consensus guidance from the senior author (L.Z.).

**4. Multidimensional Evidence Matrix Operationalization**

Included studies were synthesized into Table 1 using a four-tier operational rubric to strictly differentiate observational correlation from preclinical causality:

**Viability & State:** Distinguishing viable, metabolically active bacteria (culture-proven, transcriptomics) from non-viable DNA fragments (16S/metagenomic detection alone).

**Mechanistic Depth:** Categorizing molecular rigor (receptor-ligand binding, isogenic knockout rescue models, *in vitro* cellular assays).

**Evidence of Oral Origin:** Classifying transmission as Confirmed, Probable (shared lineages / anatomical contiguity), Associative (swallowing/aspiration pathways), or Primary Extra-oral (indigenous gut/urogenital taxa).

**Evidentiary Domain Bounding:** Explicitly restricting causal conclusions to their demonstrated experimental domains (*in vitro*, murine models) to avoid over-extrapolation to human pathogenesis.
