# Supplementary material for: Functional heterogeneity and health impacts of oral bacterial ectopic colonization
Source: Front Microbiol. 2026 Sep 25;17:1915341. doi: 10.3389/fmicb.2026.1915341 (PMC13650913; doi:10.3389/fmicb.2026.1915341)
Supplement: Supplementary file 2 [file Supplementary_file_2.DOCX]

| **Supplementary Table S2**. **Comparative Overview and Evidentiary Evaluation of Common Oral Bacteria in Systemic Diseases.** | | | | | | | |
| --- | --- | --- | --- | --- | --- | --- | --- |
| **Taxon** | **Disease/Homeostasis Context** | **Colonization Site** | **Proposed Mechanisms** | **Ectopic Colonization** | **Evidence of Oral Origin** | **Evidence Basis** | **Limitations** |
| ***Streptococcus salivarius*** | Allergic Rhinitis (AR) Exacerbation | Nasal cavity | Adheres to nasal epithelial cells by binding to Mucin5AC protein; upregulates inflammation-related genes (e.g., IL-1β, IL-6, TNF-α)(Miao et al., 2023). | Active Pathogenicity | Probable (genomic / anatomical contiguity) | **Viability & State:** Culture-proven  **Mechanistic Depth:** Receptor-ligand binding (Mucin5AC adherence)  **Interventional Validation:** Murine intervention (*in vivo* AR mouse model)  **Human Evidence:** Cross-sectional (nasal cohort 16S rRNA profiling)(Miao et al., 2023) | Rodent nasal mucosa differs immunologically from humans; clinical efficacy of targeted intervention lacks large-scale human validation. |
|  | Childhood Malnutrition & Stunting | Intestine (Small intestine /Duodenum) | Secretes low-molecular-weight products that reduce intestinal lipid absorption, disrupt the epithelial barrier, and induce small intestinal inflammation(Vonaesch et al., 2022). | Active Pathogenicity | Confirmed (paired strain-level metagenomics) | **Viability & State:** Culture-proven (duodenal aspirates)  **Mechanistic Depth:** *In vitro* assay (enterocyte lipid transport inhibition)  **Interventional Validation:** Gnotobiotic animal model (germ-free mice reproducing stunting)  **Human Evidence:** Prospective cohort (Afribiota pediatric clinical study) (Vonaesch et al., 2022) | The precise molecular identities of the secreted low-molecular-weight products and their direct host receptors are still being fully characterized. |
|  | Crohn's Disease Activity | Duodenum, stomach, intestine | Actively colonizes and adapts to the intestinal microenvironment(Hu et al., 2021). | Stable Colonization | Confirmed (paired strain-level tracking) | **Viability & State:** Longitudinal persistence  **Mechanistic Depth:** Metagenomic strain-tracking & clinical relative abundance profiling  **Interventional Validation:** None  **Human Evidence:** Cross-sectional (matched oral-gut clinical metagenomics)(Hu et al., 2021) | Evidence is primarily observational; whether *S. salivarius* actively drives Crohn's flares or is merely an opportunistic bystander remains unclear. |
| ***Streptococcus oralis*** | Commensal Homeostasis | Oral mucosa, hard palate, keratinized gingiva | Suppresses NF-κB signaling to inhibit CXCL8 (IL-8) secretion, thereby modulating mucosal immune responses and maintaining homeostasis(Myers et al., 2021). | Passive Detection / Associative | Confirmed (core resident commensal) | **Viability & State:** Culture-proven (native oral biofilm)  **Mechanistic Depth:** *In vitro* assay (NF-$\kappa$B pathway inhibition in epithelial co-cultures)  **Interventional Validation:** None  **Human Evidence:** *In vitro* assay (primary/cell line assays using clinical plaque isolates) (Myers et al., 2021) | Entirely demonstrated *in vitro*; lacks robust *in vivo* homeostatic models to prove direct suppression of systemic or mucosal inflammation in humans. |
|  | Opportunistic Mucosal Invasion (Synergistic) | Oral cavity, intestine | Synergistically forms co-aggregates with *Candida albicans*, activating host μ-calpain to degrade E-cadherin at epithelial junctions, facilitating tissue invasion and deep organ dissemination(Diaz et al., 2012; Xu et al., 2016). | Active Pathogenicity | Confirmed (core oral commensal pathobiont) | **Viability & State:** Culture-proven (co-aggregated invasive biofilm)  **Mechanistic Depth:** Receptor-ligand binding / *In vitro* assay ($\mu$-calpain activation & E-cadherin cleavage)  **Interventional Validation:** Murine intervention (*in vivo* oral co-infection & organotypic models)  **Human Evidence:** Cross-sectional (mucosal dysbiosis correlation)(Diaz et al., 2012; Xu et al., 2016) | Cooperative invasion and calpain activation are validated in mice, but translating these inter-kingdom interactions to human mucosal disease requires direct clinical observation. |
|  | Gut-Bladder Axis & Bladder Pathogen | Intestine (affecting Bladder health) | In mono-colonized germ-free mice, actively colonizes the intestinal tract and experimentally induces systemic oxidative stress and fibrotic responses in bladder tissue(Nakamura et al., 2025). | Stable Colonization (Validated in Gnotobiotic Rodent Models) | Associative (translocated via swallowing) | **Viability & State:** Culture-proven (viable gut colonization)  **Mechanistic Depth:** *In vitro* assay (fibroblast activation & oxidative stress assays)  **Interventional Validation:** Gnotobiotic animal model (mono-colonization of germ-free mice reproducing bladder fibrosis)  **Human Evidence:** None (clinical cohort correlation pending)(Nakamura et al., 2025) | Currently validated in specific mouse models; clinical correlation or cohort study validation in human cystitis patients is pending. |
| ***Streptococcus anginosus*** | Gastric Cancer Progression & Tumorigenesis | Gastric mucosa | In murine and cellular models, surface lipoprotein TmpC directly engages ANXA2 to trigger MAPK signaling, while bacterial MetE-driven methionine synthesis mechanistically stimulates tumor proliferation *in vitro* and in mice(Zhou et al., 2026). | Active Pathogenicity | Probable (genomic / shared oral-gastric lineage) | **Viability & State:** Active metabolic adaptation (methionine biosynthesis)  **Mechanistic Depth:** Isogenic mutant (KO/rescue) ($\Delta metE$ knockout) & Receptor-ligand binding (TmpC-ANXA2 direct binding)  **Interventional Validation:** Gnotobiotic animal model (germ-free & conventional mice reproducing gastric dysplasia & cancer)  **Human Evidence:** Prospective cohort / Cross-sectional (significant mucosal enrichment in clinical GC cohorts)(Fu et al., 2024; Zhou et al., 2026) | Although animal data are highly robust, the synergistic pathogenic interaction with concurrent *H. pylori* infection in humans requires further clinical longitudinal studies. |
|  | Cardiovascular Stroke Risk Prediction | Intestine | Translocates to and is enriched in the gut of stroke patients; serves as a strong predictor of 2-year adverse cardiovascular outcomes(Tonomura et al., 2025). | Passive Detection / Associative | Probable (oral-to-gut translocation) | **Viability & State:** DNA detection only (fecal 16S rRNA / metagenomic profiling)  **Mechanistic Depth:** None (mechanistic causation of gut-derived vascular injury unverified)  **Interventional Validation:** None  **Human Evidence:** Prospective cohort (clinical stroke cohort with 2-year longitudinal follow-up) | Currently restricted to clinical predictive association; the precise mechanistic link between gut *S. anginosus* expansion and acute myocardial/cerebral events is unclear. |
| ***Streptococcus sanguinis*** | Infective Endocarditis (IE) | Bloodstream / Heart valves | Enters bloodstream via transient bacteremia; utilizes Type IV Pili (PilB/PilT/PilC) and surface adhesins to bind platelets and adhere to vascular endothelial cells, initiating vegetation formation(Martini et al., 2021; Raynaud et al., 2021). | Active Pathogenicity | Confirmed (paired clinical oral & cardiac valve isolates) | **Viability & State:** Culture-proven (persistent cardiac vegetations)  **Mechanistic Depth:** Isogenic mutant (KO/rescue) (isogenic pilus mutants) & Receptor-ligand binding (platelet binding assays)  **Interventional Validation:** Murine intervention (rabbit & mouse experimental infective endocarditis models)  **Human Evidence:** Cross-sectional (clinical IE isolate genomic profiling & diagnostic blood cultures)(Baker et al., 2019; Martini et al., 2021; Raynaud et al., 2021) | Although its adherence and pilus genetics are fully mapped, prevention efficacy in clinical populations (e.g., antibiotic prophylaxis) remains a subject of ongoing epidemiological debate. |
| ***Streptococcus mitis*** | Intrauterine Infection & Placental Colonization | Placenta | Enters placental blood vessels via transient bacteremia and adheres to vascular endothelial cells, exploiting viridans group streptococci adhesin-host interactions to cross placental barriers(Fardini et al., 2010). | Active Pathogenicity | Probable | **Viability & State:** Culture-proven (viable bacterial isolation from placenta/amniotic fluid)  **Mechanistic Depth:** Receptor-ligand binding / *In vitro* assay (endothelial adherence assays)  **Interventional Validation:** Murine intervention (intravenous inoculation in pregnant mouse models causing intrauterine infection) (Fardini et al., 2010)  **Human Evidence:** Cross-sectional (PCR/metagenomic detection in human clinical placenta) | Direct tracking of oral-to-placental translocation in clinical human cohorts is ethically restricted; mechanisms rely heavily on animal models. |
| ***Veillonella parvula*** | Oral Plaque Synergism & Caries | Oral cavity (Dental plaque) | Ferments organic acids (utilizes lactic acid produced by *streptococci*)(Rogosa, 1964; Delwiche et al., 1985); enhances plaque biofilm maturation and acid resistance(Wei et al., 2024). | Passive Detection / Associative | Confirmed (core resident oral taxon) | **Viability & State:** Culture-proven (active metabolic lactate consumption)  **Mechanistic Depth:** *In vitro* assay (multi-species biofilm co-cultures with *S. mutans*, peroxide detoxification)  **Interventional Validation:** None  **Human Evidence:** Cross-sectional (plaque metagenomic profiling from severe caries cohorts)(Rogosa, 1964; Delwiche et al., 1985; Wei et al., 2024) | Synergistic metabolic interactions are highly niche-dependent and rely on strict local nutrient availability. |
|  | Ectopic Intestinal Colonization (Colitis) | Gastrointestinal tract (Intestine) | Undergoes a metabolic shift from fermentation to anaerobic nitrate respiration via the nar gene cluster (specifically *narG*), using microenvironmental nitrite for rapid proliferation(Rojas-Tapias et al., 2022). | Stable Colonization | Probable (oral-to-gut translocation) | **Viability & State:** Active metabolic adaptation (nitrate respiration switch)  **Mechanistic Depth:** Isogenic mutant (KO/rescue) (isogenic $\Delta narG$ mutant loses colonization fitness)  **Interventional Validation:** Murine intervention (DSS colitis mouse models & host $iNOS^{-/-}$ knockout validation)  **Human Evidence:** Cross-sectional (enrichment in IBD patient metagenomic cohorts) (Rojas-Tapias et al., 2022) | Primarily studied in the context of active inflammation (colitis); the threshold of inflammation and nitrite required to trigger this metabolic shift in a healthy or sub-clinically inflamed human gut is unclear. |
| ***Veillonella dispar*** | Dental Caries Susceptibility | Oral cavity (Dental plaque) | High oral abundance combined with dietary/nutritional risk correlates with caries status in children(Lee et al., 2025). | Passive Detection / Associative | Confirmed (resident oral commensal) | **Viability & State:** Culture-proven (salivary & plaque detection)  **Mechanistic Depth:** None (specific cariogenic mechanism undefined)  **Interventional Validation:** None  **Human Evidence:** Cross-sectional (pediatric clinical cohort correlating oral abundance with caries index)(Lee et al., 2025) | As a lactate-consuming commensal, the specific pathogenic role of V. dispar in caries development remains unclear; lacks causal *in vivo* validation. |
|  | Ulcerative Colitis (UC) Correlation | Intestine | Ectopically colonizes and expands in the inflamed gut (Atarashi et al., 2017); its abundance positively correlates with mucosal inflammation and disease course (Atarashi et al., 2017; Rojas-Tapias et al., 2022). | Passive Detection / Associative | Probable (translocated oral commensal) | **Viability & State:** DNA detection only (fecal/mucosal 16S rRNA & metagenomic detection)  **Mechanistic Depth:** None (lacks species-specific isogenic mutant validation)  **Interventional Validation:** None  **Human Evidence:** Cross-sectional (clinical profiling of treatment-naïve UC cohorts)(Atarashi et al., 2017; Rojas-Tapias et al., 2022) | No specific causal *in vivo* model exists for *V. dispar* in UC; previous claims of nitrate-respiration causality belong strictly to *V. parvula*. |
| ***Veillonella rogosae*** | IBD Progression & Gut Inflammation | Intestine | Oral-to-gut translocation; elevated abundance positively correlates with gut inflammation and fecal calprotectin levels(Rocha et al., 2023). | Passive Detection / Associative | Probable (Genomic/Eco) | **Viability & State:** DNA detection only (fecal metagenomic sequencing)  **Mechanistic Depth:** None (molecular link between diet, colonization, and epithelial damage uncharacterized)  **Interventional Validation:** None  **Human Evidence:** Cross-sectional (clinical cohort correlating patient dietary indices, metagenomics, and calprotectin)(Rocha et al., 2023) | Lacks direct validation in gnotobiotic or gene-knockout animal models; the causal chain from pro-inflammatory diet to *V. rogosae*-driven colitis is unproven. |
| ***Veillonella atypica*** | Oral-Gut-Myositis Axis Disruption | Intestine | Enriched in the fecal metagenome of myositis patients; statistically correlated with disease activity markers, but direct mucosal barrier disruption remains unproven experimentally (Li et al., 2023). | Passive Detection / Associative | Probable (oral commensal translocated to gut) | **Viability & State:** DNA detection only (metagenome-wide association sequencing)  **Mechanistic Depth:** None (specific molecular mechanism triggering muscle autoimmunity undefined)  **Interventional Validation:** None  **Human Evidence:** Cross-sectional (metagenome-wide association study in myositis cohorts)(Li et al., 2023) | Primarily based on metagenomic association; the exact molecular mechanism of how *V. atypica* translocation modulates systemic muscle autoimmunity is unknown. |
| ***Prevotella intermedia*** | Severe Bacteremic Pneumococcal Pneumonia | Respiratory tract | Adheres to respiratory epithelial cells (e.g., A549) via surface adhesins; uniquely upregulates PAFR transcription to facilitate *S. pneumoniae* invasion, leading to systemic bacteremia and cytokine storms(Nagaoka et al., 2014; Wu et al., 2021). | Active Pathogenicity | Confirmed (resident oral pathobiont translocated via microaspiration) | **Viability & State:** Culture-proven (viable respiratory co-colonization)  **Mechanistic Depth:** Receptor-ligand binding / *In vitro* assay (upregulation of host epithelial PAFR expression)  **Interventional Validation:** Murine intervention (*in vivo* co-infection models demonstrating increased lung/blood bacterial loads & mortality) (Nagaoka et al., 2014; Wu et al., 2021)  **Human Evidence:** Cross-sectional (airway aspiration & microbial co-occurrence profiling) | Most mechanistic steps of PAFR upregulation are characterized in murine systems; clinical verification of this synergism in human pneumococcal cohorts is limited. |
|  | Duodenal Colonization & Childhood Stunting | Intestine (Duodenum) | Detected as a core member of ectopically colonizing flora in the small intestine, disrupting local nutrient absorption and correlating with enteropathy(Chen et al., 2020). | Active Pathogenicity | Confirmed (paired oral-duodenal metagenomic tracking) | **Viability & State:** Culture-proven (viable duodenal aspirates from stunted children)  **Mechanistic Depth:** *In vitro* assay (enterocyte barrier disruption & inflammatory activation)  **Interventional Validation:** Gnotobiotic animal model (germ-free mice colonized with patient consortia reproducing enteropathy & stunting)  **Human Evidence:** Prospective cohort (NEJM clinical cohort of undernourished children)(Chen et al., 2020) | Long-term intervention trials are required to prove whether eradicating duodenal *P. intermedia* can reverse child stunting. |
| ***Prevotella histicola*** | Colitis Alleviation & Immune Regulation | Intestine | Demonstrated in murine DSS-induced colitis models: orally administered strains suppress mucosal IRE1α-JNK endoplasmic reticulum stress and NF-κB signaling, exerting anti-inflammatory effects (Fan et al., 2024). | Active Host Modulation | Confirmed (Human oral mucosal isolate) | **Viability & State:** Culture-proven (viable oral gavage transit & colonization)  **Mechanistic Depth:** *In vitro* assay (cellular ER stress & NF-$\kappa$B inhibition assays)  **Interventional Validation:** Murine intervention (*in vivo* DSS-induced colitis mouse model proving tissue recovery & cytokine suppression) (Fan et al., 2024)  **Human Evidence:** None (preclinical animal validation; human biotherapeutic clinical trials pending) | Primarily validated in rodent models of acute/chronic colitis; human clinical trials utilizing *P. histicola* as an oral-derived biotherapeutic are still in early stages. |
| ***Lactobacillus rhamnosus*** | Systemic Bacteremia & Sepsis | Bloodstream / Endocardium | Disseminates hematogenously to extra-oral/extra-intestinal sites, potentially leading to opportunistic bacteremia or endocarditis(Ioannou et al., 2024). | Passive Detection / Associative | Primary Extra-oral | **Viability & State:** Culture-proven (clinical diagnostic blood cultures)  **Mechanistic Depth:** None (opportunistic barrier breach lacking active host-targeted virulence machinery)  **Interventional Validation:** None  **Human Evidence:** Case report (clinical case series, retrospective reviews, and pharmacovigilance safety analyses)(Ioannou et al., 2024) | Highly strain-dependent; translocation is extremely rare in immunocompetent hosts and lacks an active host-targeted molecular pathobiont pathway. |
|  | Respiratory Protection | Respiratory mucosa | Modulates immune response via the TLR4/MyD88/NF-$\kappa$B pathway ; reduces lung IL-5 and eotaxin production to limit allergic hyperreactivity(Wu et al., 2022b). | Active Host Modulation | Primary Extra-oral | **Viability & State:** Culture-proven (viable suspension)  **Mechanistic Depth:** *In vitro* assay (TLR4/MyD88/NF-$\kappa$B pathway modulation)  **Interventional Validation:** Murine intervention (*in vivo* OVA-LPS asthma mouse model) (Wu et al., 2022b)  **Human Evidence:** None (preclinical animal intervention) | Evidence is based on artificial pharmacological nasal inoculation, not natural oral-to-airway translocation. tion is limited. |
| ***Lactobacillus gasseri*** | Cholestatic Liver Disease Exacerbation | Liver | In an Mdr2−/− mouse model of leaky gut, translocated commensal lactobacilli correlated with intrahepatic γδ T-cell IL-17 activation (Tedesco et al., 2018); acts as an opportunistic trigger in compromised mucosal barriers rather than a primary pathogen. | Passive Detection / Associative | Primary Extra-oral | **Viability & State:** Culture-proven (viable extra-intestinal hepatic translocation)  **Mechanistic Depth:** Translocated bacterial cell components stimulate TLRs on intrahepatic $\gamma\delta$ T cells to induce IL-17 secretion and promote biliary fibrosis in Mdr2−/−mice  **Interventional Validation:** Murine intervention (*in vivo* $\mathrm{Mdr}2^{-/-}$ PSC mouse model)(Tedesco et al., 2018)  **Human Evidence:** Cross-sectional (gut dysbiosis association in PSC cohorts) | Causality is established in mouse models, but direct clinical evidence showing oral-derived *L. gasseri* driving human PSC progression is limited. |
|  | Periodontal Antioxidant Protection | Periodontal tissues | Upregulates the Akt-Nrf2-HO-1 signaling pathway to mitigate oxidative stress and periodontal inflammation(Lee et al., 2023). | Active Host Modulation | Confirmed (resident oral/mucosal lactic acid bacterium) | **Viability & State:** Culture-proven (culture media / probiotic extract)  **Mechanistic Depth:** *In vitro* assay (Akt-Nrf2-HO-1 pathway activation in gingival fibroblasts)  **Interventional Validation:** Murine intervention (ligature-induced periodontitis mouse model)(Lee et al., 2023)  **Human Evidence:** None (preclinical formulation testing) | Tested primarily as an exogenous probiotic media formulation; the endogenous protective role of resident oral strains needs further clinical proof. |
| ***Lactobacillus plantarum*** | Systemic Opportunistic Infection | Bloodstream | Potential hematogenous translocation leading to systemic infection in susceptible/vulnerable hosts(Kullar et al., 2023; Ioannou et al., 2024). | Passive Detection / Associative | Primary Extra-oral | **Viability & State:** Culture-proven (isolated from clinical blood cultures)  **Mechanistic Depth:** None (passive opportunistic entry lacking specific host-targeted virulence machinery)  **Interventional Validation:** None  **Human Evidence:** Case report (systematic reviews and pharmacovigilance safety analyses in immunocompromised patients)(Kullar et al., 2023; Ioannou et al., 2024) | Occurs almost exclusively in hosts with extreme immunological or mucosal barrier deficits; no specific molecular translocation pathway is established. |
| ***Helicobacter pylori*** | Periodontitis & Oral Reservoir | Oral cavity (Dental plaque) | Colonizes dental plaque (extragastric reservoir)(Cuba et al., 2025); alters the microecology of periodontal tissues and increases local IL-17 levels(Fan et al., 2025). | Passive Detection / Associative | Primary Extra-oral | **Viability & State:** DNA detection only (subgingival / salivary PCR detection; permanent viability debated)  **Mechanistic Depth:** *In vitro* assay (correlative cytokine shifts and subgingival dysbiosis)  **Interventional Validation:** None (lacks *in vivo* oral periodontitis induction models)  **Human Evidence:** Cross-sectional (systematic reviews & NHANES III / clinical periodontal cohort PCR detection)(Cuba et al., 2025; Fan et al., 2025) | The causal direction of whether oral *H. pylori* directly induces periodontitis or simply exploits the inflamed pocket remains controversial. |
|  | Gastric Mucosa Disruption & Carcinogenesis | Gastric mucosa | Secretes virulence factors CagA and VacA; CagA integrates into host cells, disrupting the gastric mucosal barrier and activating oncogenic pathways(Saju et al., 2016). | Active Pathogenicity | Primary Extra-oral | **Viability & State:** Active metabolic adaptation (urease-driven acid survival)  **Mechanistic Depth:** Isogenic mutant (KO/rescue) (isogenic CagA mutants) & Receptor-ligand binding (CagA-SHP2)  **Interventional Validation:** Clinical trial (randomized eradication trials reversing lesions & MALT lymphoma)  **Human Evidence:** Clinical interventional RCT (decades of human clinical cohorts & trials)(Saju et al., 2016; Salar, 2019) | Decades of clinical RCTs confirm gastric pathogenesis, but direct *in vivo* tracking proving that extra-gastric oral reservoirs are the primary initiator of recurrent gastric infection remains technically challenging. |
|  | Systemic Metabolic & Iron Dysfunction | Duodenum / Intestine | Inhibits hepatic metabolic function and insulin signaling(Liu et al., 2023). Disrupts duodenal iron absorption, leading to iron-deficiency anemia (IDA)(Kato et al., 2022). | Active Pathogenicity | Primary Extra-oral | **Viability & State:** Culture-proven (duodenal & gastric mucosal colonization)  **Mechanistic Depth:** *In vitro* assay (iron transport inhibition & cytokine release)  **Interventional Validation:** Clinical trial (antibiotic eradication therapy in pediatric IDA patients restoring ferritin)  **Human Evidence:** Clinical interventional RCT / Prospective cohort (pediatric clinical trials & meta-analyses)(Gonciarz et al., 2020; Kato et al., 2022) | The severity of systemic metabolic impairment depends strongly on host genetics and co-morbidities. |
| ***Fusobacterium nucleatum*** | Colorectal Cancer (CRC) Enrichment | Gastrointestinal tract (Gut tumor microenvironment) | Fap2 adhesin binds to Gal-GalNAc on tumor surfaces(Abed et al., 2016). FadA binds E-cadherin to activate $\beta$-catenin(Rubinstein et al., 2013).  Mechanistically demonstrated in murine colitis models: outer vesicles/bacteria provoke epithelial RIPK1-dependent necrosome assembly and lipid-peroxidation-driven ferroptosis (Zhang et al., 2025) and RIPK1-mediated death(Liu et al., 2021). | Active Pathogenicity | Confirmed (paired oral-colorectal metagenomic tracking) | **Viability & State:** Active metabolic adaptation (tumor niche survival & metabolic rewiring)  **Mechanistic Depth:** Isogenic mutant (KO/rescue) ($\Delta fadA,\Delta fap2$ knockouts) & Receptor-ligand binding (FadA-E-cadherin, Fap2-Gal-GalNAc)  **Interventional Validation:** Murine intervention (mouse xenograft models, $Apc^{min/+}$ mice & metronidazole response)  **Human Evidence:** Cross-sectional / Prospective cohort (human CRC cohort metagenomics & tumor biopsies)(Fardini et al., 2011; Abed et al., 2016; Bullman et al., 2017; Liu et al., 2021; Zhang et al., 2025) | The contribution of oral translocated strains versus endogenous gut strains during the very early stages of CRC initiation is still being dissected. |
|  | Adverse Pregnancy Outcomes & Preterm Birth | Placenta | Fap2 binds Gal-GalNAc on placental cells(Coppenhagen-Glazer et al., 2015). FadA adheres to endothelial cells, disrupting VE-cadherin to increase vascular permeability(Fardini et al., 2011). | Active Pathogenicity | Confirmed (paired maternal subgingival & placental strain concordance) | **Viability & State:** Culture-proven (viable bacterial isolation from placenta/amniotic fluid)  **Mechanistic Depth:** Isogenic mutant (KO/rescue) ($\Delta fap2$ and $\Delta fadA$ mutants) & Receptor-ligand binding (VE-cadherin disruption)  **Interventional Validation:** Murine intervention (pregnant mouse transmission models causing fetal loss)  **Human Evidence:** Cross-sectional (human placental PCR + clinical cohort association)(Fardini et al., 2010; Coppenhagen-Glazer et al., 2015; Xu and Han, 2022; Kotzur et al., 2024) | Human placental models are ethically limited; *in vivo* studies rely on pregnant rodent models which differ in placental architecture. |
|  | Periodontitis & Renal Fibrosis Promotion | Extracellular vesicle (OMV) / Circulatory dissemination (Non-colonizing systemic effect) | OMVs enhance periodontal inflammation and bone resorption(Zhang et al., 2024). Shown in a rat ligation-periodontitis model: translocated components experimentally upregulate TGF-β/SMAD2/3 signaling in renal tubular cells(Chen et al., 2024). | Active Pathogenicity | Confirmed (core periodontal pathogen) | **Viability & State:** Culture-proven / OMV shedding  **Mechanistic Depth:** *In vitro* assay (renal tubular epithelial EMT assays & pathway activation)  **Interventional Validation:** Murine intervention (*in vivo* rat periodontitis-renal fibrosis models)(Chen et al., 2024; Zhang et al., 2024)  **Human Evidence:** None (preclinical animal intervention) | Translational evidence linking periodontal *F. nucleatum* directly to chronic kidney disease in human clinical cohorts is still emerging. |
| ***Porphyromonas gingivalis*** | Alzheimer’s Disease & Neuroinflammation | Brain | In rodent experimental systems, EVs translocate via trigeminal/vascular routes to evoke microglial activation and memory deficits; human evidence is currently restricted to post-mortem tissue detection and clinical association(Owoyele and Malekzadeh, 2022; Ma et al., 2023). | Active Pathogenicity | Confirmed (core periodontal keystone pathogen) | **Viability & State:** Culture-proven / EV neural translocation  **Mechanistic Depth:** *In vitro* assay (microglial pro-inflammatory activation & gingipain neurotoxicity)  **Interventional Validation:** Murine intervention (*in vivo* oral ligation models & EV intrathecal injection inducing cognitive deficits)  **Human Evidence:** Cross-sectional (post-mortem human brain PCR & clinical cohort association)(Owoyele and Malekzadeh, 2022; Ma et al., 2023) | BBB penetration and trigeminal pathways are primarily confirmed in rodents; clinical intervention trials targeting brain gingipains in humans have yielded mixed therapeutic outcomes. |
|  | MASLD / Liver Injury Progression | Liver | Surface components (fimbriae, OmpA, LPS) promote MASLD via the CD36-PPAR𝛾 signaling pathway, inducing macrophage polarization, hepatic inflammation, and lipid dysregulation(Ahn et al., 2021; Wu et al., 2022a). | Active Pathogenicity | Confirmed (core periodontal pathogen) | **Viability & State:** Active metabolic adaptation (upregulates BCAA-transfer genes *livH/livK*)  **Mechanistic Depth:** Dual RNA-seq & host BCAA metabolic profiling (transcriptional upregulation of *livH/livK*)  **Interventional Validation:** Murine intervention (high-fat diet mouse models with oral P. gingivalis gavage exacerbating liver injury)  **Human Evidence:** Cross-sectional (clinical MASLD/NAFLD cohort correlation with periodontitis)(Ahn et al., 2021; Wu et al., 2022a) | Relies heavily on synergistic animal models (diet + infection); direct causal contribution of oral Pg to clinical human liver pathology requires further longitudinal studies. |
|  | Adverse Pregnancy Outcomes | Placenta | Translocates via transient bacteremia; secretes gingipain enzymes to disrupt epithelial junctions(Katz et al., 2002; Takeuchi et al., 2019) and upregulates cytokines (IFN-𝛾, IL-2) in placental tissues(Lin et al., 2003). | Active Pathogenicity | Confirmed | **Viability & State:** Culture-proven in animal placenta; DNA detection in human placenta. **Mechanistic Depth:** Gingipain degradation of endothelial junctions & placental cytokine upregulation (IFN-$\gamma$, IL-2) **Interventional Validation:** Murine pregnant transmission models causing fetal loss **Human Evidence:** Cross-sectional placental PCR/metagenomic detection (Lin et al., 2003; Barak et al., 2007), human periodontal intervention RCTs show mixed/limited preterm reduction. | The clinical intervention paradox exists: although animal causality is robust, large-scale human RCTs (e.g., the OPT study) show that periodontal therapy during pregnancy does not significantly reduce preterm birth rates. |
| ***Tannerella forsythia*** | Atherosclerosis Progression | Vascular wall / Carotid plaque | Adheres via surface glycoproteins (SiaH, NanH); BspA protein triggers TLR2/MyD88 signaling to induce foam cell formation in macrophages (THP-1) and promote plaque maturation(Sharma, 2010; Lee et al., 2014). | Active Pathogenicity | Confirmed (core "red complex" periodontal pathogen) | **Viability & State:** DNA detection only in human atheroma (metagenomics/PCR); culture-based challenge in ApoE^-/-^ mouse models  **Mechanistic Depth:** Receptor-ligand binding (BspA-TLR2 binding) & *In vitro* assay (macrophage THP-1 foam cell formation & methylglyoxal stress)  **Interventional Validation:** Murine intervention (*in vivo* $\mathrm{Apo}E^{-/-}$ atherosclerotic mouse models)  **Human Evidence:** Cross-sectional (16S rRNA / metagenomic detection of bacterial DNA in carotid plaques)(Rao et al., 2023) | Plaque colonization is largely validated in animal acceleration models; direct causal contribution to clinical plaque rupture in humans remains correlative. |
|  | Systemic Metabolic Dysregulation | Oral mucosa / Systemic circulation | Observed in an adult catch-up fat rat model: tongue coating inoculation was associated with enhanced high-fat appetite and aggravated insulin resistance(Hu et al., 2018). | Passive Detection / Associative | Confirmed (tongue dorsum resident anaerobe) | **Viability & State:** Culture-proven (dorsal tongue biofilm colonization)  **Mechanistic Depth:** *In vitro* assay (correlative shifts in high-fat food preference and peripheral insulin sensitivity)  **Interventional Validation:** Murine intervention (diet-induced obese rat model with dorsal tongue inoculation)  **Human Evidence:** Cross-sectional (human dietary cohorts associating tongue coating abundance with visceral fat accumulation)(Hu et al., 2018) | Mostly correlative in humans (tongue coating and metabolic syndrome); direct *in vivo* mechanisms linking its oral abundance to peripheral insulin resistance are still emerging. |

This table applies a multidimensional evidentiary matrix to evaluate oral bacterial ectopic colonization and systemic disease associations. **Ectopic Colonization** differentiates *Passive Detection / Associative* (DNA detection or transient transit), *Stable Colonization* (viable persistence or metabolic adaptation), *Active Pathogenicity* (experimentally demonstrated tissue invasion, receptor engagement, or oncometabolite production in model systems), and *Active Host Modulation* (preclinical protective/probiotic immune regulation). **Evidence of Oral Origin** distinguishes *Confirmed*, *Probable* (shared lineages or anatomical contiguity), *Associative* (general translocation routes), and *Primary Extra-oral* (indigenous gut/urogenital taxa). The **Evidence Basis** explicitly separates preclinical experimental systems (*in vitro*, gnotobiotic/murine models) from human clinical cohorts to prevent overstating experimental causality as established human pathogenesis.

References

Abed, J., Emgård, J. E. M., Zamir, G., Faroja, M., Almogy, G., Grenov, A., et al. (2016). Fap2 Mediates Fusobacterium nucleatum Colorectal Adenocarcinoma Enrichment by Binding to Tumor-Expressed Gal-GalNAc. *Cell Host Microbe* 20, 215–225. doi: 10.1016/j.chom.2016.07.006

Ahn, J.-S., Yang, J. W., Oh, S.-J., Shin, Y. Y., Kang, M.-J., Park, H. R., et al. (2021). Porphyromonas gingivalis exacerbates the progression of fatty liver disease via CD36-PPARγ pathway. *BMB Rep* 54, 323–328. doi: 10.5483/BMBRep.2021.54.6.050

Atarashi, K., Suda, W., Luo, C., Kawaguchi, T., Motoo, I., Narushima, S., et al. (2017). Ectopic colonization of oral bacteria in the intestine drives TH1 cell induction and inflammation. *Science* 358, 359–365. doi: 10.1126/science.aan4526

Baker, S. P., Nulton, T. J., and Kitten, T. (2019). Genomic, Phenotypic, and Virulence Analysis of Streptococcus sanguinis Oral and Infective-Endocarditis Isolates. *Infect Immun* 87, e00703-18. doi: 10.1128/IAI.00703-18

Barak, S., Oettinger-Barak, O., Machtei, E. E., Sprecher, H., and Ohel, G. (2007). Evidence of periopathogenic microorganisms in placentas of women with preeclampsia. *J Periodontol* 78, 670–676. doi: 10.1902/jop.2007.060362

Bullman, S., Pedamallu, C. S., Sicinska, E., Clancy, T. E., Zhang, X., Cai, D., et al. (2017). Analysis of Fusobacterium persistence and antibiotic response in colorectal cancer. *Science* 358, 1443–1448. doi: 10.1126/science.aal5240

Chen, P., Lin, X., Zhang, C., Xie, Y., Guo, Z., and Ren, F. (2024). Fusobacterium nucleatum-infected periodontitis promotes renal interstitial fibrosis in rats through the TGF-β/SMAD2/3 and β-catenin signaling pathways. *Gene* 927, 148729. doi: 10.1016/j.gene.2024.148729

Chen, R. Y., Kung, V. L., Das, S., Hossain, Md. S., Hibberd, M. C., Guruge, J., et al. (2020). Linking the duodenal microbiota to stunting in a cohort of undernourished Bangladeshi children with enteropathy. *N Engl J Med* 383, 321–333. doi: 10.1056/NEJMoa1916004

Coppenhagen-Glazer, S., Sol, A., Abed, J., Naor, R., Zhang, X., Han, Y. W., et al. (2015). Fap2 of Fusobacterium nucleatum Is a Galactose-Inhibitable Adhesin Involved in Coaggregation, Cell Adhesion, and Preterm Birth. *Infect Immun* 83, 1104–1113. doi: 10.1128/IAI.02838-14

Cuba, E., Sánchez, M. C., Ciudad, M. J., and Collado, L. (2025). Association of Helicobacter pylori as an Extragastric Reservoir in the Oral Cavity with Oral Diseases in Patients with and Without Gastritis-A Systematic Review. *Microorganisms* 13, 1955. doi: 10.3390/microorganisms13081955

Delwiche, E. A., Pestka, J. J., and Tortorello, M. L. (1985). The veillonellae: gram-negative cocci with a unique physiology. *Annu Rev Microbiol* 39, 175–193. doi: 10.1146/annurev.mi.39.100185.001135

Diaz, P. I., Xie, Z., Sobue, T., Thompson, A., Biyikoglu, B., Ricker, A., et al. (2012). Synergistic Interaction between Candida albicans and Commensal Oral Streptococci in a Novel In Vitro Mucosal Model. *Infect Immun* 80, 620–632. doi: 10.1128/IAI.05896-11

Fan, X., Lu, Q., Jia, Q., Li, L., Cao, C., Wu, Z., et al. (2024). Prevotella histicola ameliorates DSS-induced colitis by inhibiting IRE1α-JNK pathway of ER stress and NF-κB signaling. *Int Immunopharmacol* 135, 112285. doi: 10.1016/j.intimp.2024.112285

Fan, Y., Chen, X., Shan, T., Wang, N., Han, Q., Ren, B., et al. (2025). Polymicrobial interactions of Helicobacter pylori and its role in the process of oral diseases. *J Oral Microbiol* 17, 2469896. doi: 10.1080/20002297.2025.2469896

Fardini, Y., Chung, P., Dumm, R., Joshi, N., and Han, Y. W. (2010). Transmission of diverse oral bacteria to murine placenta: evidence for the oral microbiome as a potential source of intrauterine infection. *Infect Immun* 78, 1789–1796. doi: 10.1128/IAI.01395-09

Fardini, Y., Wang, X., Témoin, S., Nithianantham, S., Lee, D., Shoham, M., et al. (2011). Fusobacterium nucleatum adhesin FadA binds vascular-endothelial cadherin and alters endothelial integrity. *Mol Microbiol* 82, 1468–1480. doi: 10.1111/j.1365-2958.2011.07905.x

Fu, K., Cheung, A. H. K., Wong, C. C., Liu, W., Zhou, Y., Wang, F., et al. (2024). Streptococcus anginosus promotes gastric inflammation, atrophy, and tumorigenesis in mice. *Cell* 187, 882-896.e17. doi: 10.1016/j.cell.2024.01.004

Gonciarz, W., Lechowicz, Ł., Urbaniak, M., Kaca, W., and Chmiela, M. (2020). Attenuated Total Reflectance Fourier Transform Infrared Spectroscopy (FTIR) and Artificial Neural Networks Applied to Investigate Quantitative Changes of Selected Soluble Biomarkers, Correlated with H. pylori Infection in Children and Presumable Consequent Delayed Growth. *J Clin Med* 9, 3852. doi: 10.3390/jcm9123852

Hu, S., Png, E., Gowans, M., Ong, D. E. H., de Sessions, P. F., Song, J., et al. (2021). Ectopic gut colonization: a metagenomic study of the oral and gut microbiome in Crohn’s disease. *Gut Pathog* 13, 13. doi: 10.1186/s13099-021-00409-5

Hu, X., Zhang, Q., Zhang, M., Yang, X., Zeng, T.-S., Zhang, J.-Y., et al. (2018). Tannerella forsythia and coating color on the tongue dorsum, and fatty food liking associate with fat accumulation and insulin resistance in adult catch-up fat. *Int J Obes (Lond)* 42, 121–128. doi: 10.1038/ijo.2017.191

Ioannou, P., Ziogou, A., Giannakodimos, I., Giannakodimos, A., Baliou, S., and Samonis, G. (2024). Infective Endocarditis by Lactobacillus Species-A Narrative Review. *Antibiotics (Basel)* 13, 53. doi: 10.3390/antibiotics13010053

Kato, S., Gold, B. D., and Kato, A. (2022). Helicobacter pylori-Associated Iron Deficiency Anemia in Childhood and Adolescence-Pathogenesis and Clinical Management Strategy. *J Clin Med* 11, 7351. doi: 10.3390/jcm11247351

Katz, J., Yang, Q.-B., Zhang, P., Potempa, J., Travis, J., Michalek, S. M., et al. (2002). Hydrolysis of epithelial junctional proteins by Porphyromonas gingivalis gingipains. *Infect Immun* 70, 2512–2518. doi: 10.1128/IAI.70.5.2512-2518.2002

Kotzur, R., Kahlon, S., Isaacson, B., Gamliel, M., Charpak-Amikam, Y., Lieberman, J., et al. (2024). Pregnancy trained decidual NK cells protect pregnancies from harmful Fusobacterium nucleatum infection. *PLoS Pathog* 20, e1011923. doi: 10.1371/journal.ppat.1011923

Kullar, R., Goldstein, E. J. C., Johnson, S., and McFarland, L. V. (2023). Lactobacillus Bacteremia and Probiotics: A Review. *Microorganisms* 11, 896. doi: 10.3390/microorganisms11040896

Lee, H. R., Jun, H. K., and Choi, B. K. (2014). Tannerella forsythia BspA increases the risk factors for atherosclerosis in ApoE(-/-) mice. *Oral Dis* 20, 803–808. doi: 10.1111/odi.12214

Lee, H.-Y., Lee, G.-H., Kim, J.-H., Cheng, J., Cho, J.-H., Suh, J.-W., et al. (2023). Ixeris dentata and Lactobacillus gasseri media protect against periodontitis through Nrf2-HO-1 signalling pathway. *Sci Rep* 13, 12861. doi: 10.1038/s41598-023-39853-5

Lee, V. H. K., de Jesus, V. C., Mittermuller, B.-A., Nickel, N. C., Chelikani, P., and Schroth, R. J. (2025). Association of Nutritional Status and Oral Veillonella With Caries Status in Preschool Children in Manitoba. *Pediatr Dent* 47, 41–49.

Li, Y., Xu, J., Hong, Y., Li, Z., Xing, X., Zhufeng, Y., et al. (2023). Metagenome-wide association study of gut microbiome features for myositis. *Clin Immunol* 255, 109738. doi: 10.1016/j.clim.2023.109738

Lin, D., Smith, M. A., Elter, J., Champagne, C., Downey, C. L., Beck, J., et al. (2003). Porphyromonas gingivalis Infection in Pregnant Mice Is Associated with Placental Dissemination, an Increase in the Placental Th1/Th2 Cytokine Ratio, and Fetal Growth Restriction. *Infect Immun* 71, 5163–5168. doi: 10.1128/IAI.71.9.5163-5168.2003

Liu, L., Liang, L., Yang, C., Zhou, Y., and Chen, Y. (2021). Extracellular vesicles of Fusobacterium nucleatum compromise intestinal barrier through targeting RIPK1-mediated cell death pathway. *Gut Microbes* 13, 1–20. doi: 10.1080/19490976.2021.1902718

Liu, Y., Shuai, P., Chen, W., Liu, Y., and Li, D. (2023). Association between Helicobacter pylori infection and metabolic syndrome and its components. *Front Endocrinol (Lausanne)* 14, 1188487. doi: 10.3389/fendo.2023.1188487

Ma, X., Shin, Y.-J., Yoo, J.-W., Park, H.-S., and Kim, D.-H. (2023). Extracellular vesicles derived from Porphyromonas gingivalis induce trigeminal nerve-mediated cognitive impairment. *J Adv Res* 54, 293–303. doi: 10.1016/j.jare.2023.02.006

Martini, A. M., Moricz, B. S., Woods, L. J., and Jones, B. D. (2021). Type IV Pili of Streptococcus sanguinis Contribute to Pathogenesis in Experimental Infective Endocarditis. *Microbiol Spectr* 9, e0175221. doi: 10.1128/Spectrum.01752-21

Miao, P., Jiang, Y., Jian, Y., Shi, J., Liu, Y., Piewngam, P., et al. (2023). Exacerbation of allergic rhinitis by the commensal bacterium Streptococcus salivarius. *Nat Microbiol* 8, 218–230. doi: 10.1038/s41564-022-01301-x

Myers, S., Do, T., Meade, J. L., Tugnait, A., Vernon, J. J., Pistolic, J., et al. (2021). Immunomodulatory streptococci that inhibit CXCL8 secretion and NFκB activation are common members of the oral microbiota. *J Med Microbiol* 70, 001329. doi: 10.1099/jmm.0.001329

Nagaoka, K., Yanagihara, K., Morinaga, Y., Nakamura, S., Harada, T., Hasegawa, H., et al. (2014). Prevotella intermedia induces severe bacteremic pneumococcal pneumonia in mice with upregulated platelet-activating factor receptor expression. *Infect Immun* 82, 587–593. doi: 10.1128/IAI.00943-13

Nakamura, N., Iioka, K., Morisaki, H., Okahashi, N., Kurosawa, M., Fukamachi, H., et al. (2025). Pathogenic effects of Streptococcus oralis intestinal colonization on bladder health in mice. *Curr Res Microb Sci* 8, 100375. doi: 10.1016/j.crmicr.2025.100375

Owoyele, P. V., and Malekzadeh, S. (2022). Porphyromonas gingivalis, neuroinflammation and Alzheimer’s disease. *Niger J Physiol Sci* 37, 157–164. doi: 10.54548/njps.v37i2.1

Rao, A., Lokesh, J., D’Souza, C., Prithvisagar, K. S., Subramanyam, K., Karunasagar, I., et al. (2023). Metagenomic Analysis to Uncover the Subgingival and Atherosclerotic Plaque Microbiota in Patients with Coronary Artery Disease. *Indian J Microbiol* 63, 281–290. doi: 10.1007/s12088-023-01082-9

Raynaud, C., Sheppard, D., Berry, J.-L., Gurung, I., and Pelicic, V. (2021). PilB from Streptococcus sanguinis is a bimodular type IV pilin with a direct role in adhesion. *Proc Natl Acad Sci U S A* 118, e2102092118. doi: 10.1073/pnas.2102092118

Rocha, I. M. G. da, Torrinhas, R., Fonseca, D., Lyra, C. de O., de Sousa Alves Neri, J. L., Balmant, B. D., et al. (2023). Pro-Inflammatory Diet Is Correlated with High Veillonella rogosae, Gut Inflammation and Clinical Relapse of Inflammatory Bowel Disease. *Nutrients* 15, 4148. doi: 10.3390/nu15194148

Rogosa, M. (1964). THE GENUS VEILLONELLA. I. GENERAL CULTURAL, ECOLOGICAL, AND BIOCHEMICAL CONSIDERATIONS. *J Bacteriol* 87, 162–170. doi: 10.1128/jb.87.1.162-170.1964

Rojas-Tapias, D. F., Brown, E. M., Temple, E. R., Onyekaba, M. A., Mohamed, A. M. T., Duncan, K., et al. (2022). Inflammation-associated nitrate facilitates ectopic colonization of oral bacterium Veillonella parvula in the intestine. *Nat Microbiol* 7, 1673–1685. doi: 10.1038/s41564-022-01224-7

Rubinstein, M. R., Wang, X., Liu, W., Hao, Y., Cai, G., and Han, Y. W. (2013). Fusobacterium nucleatum promotes colorectal carcinogenesis by modulating E-cadherin/β-catenin signaling via its FadA adhesin. *Cell Host Microbe* 14, 195–206. doi: 10.1016/j.chom.2013.07.012

Saju, P., Murata-Kamiya, N., Hayashi, T., Senda, Y., Nagase, L., Noda, S., et al. (2016). Host SHP1 phosphatase antagonizes Helicobacter pylori CagA and can be downregulated by Epstein-Barr virus. *Nat Microbiol* 1, 16026. doi: 10.1038/nmicrobiol.2016.26

Salar, A. (2019). Gastric MALT lymphoma and Helicobacter pylori. *Med Clin (Barc)* 152, 65–71. doi: 10.1016/j.medcli.2018.09.006

Sharma, A. (2010). Virulence mechanisms of Tannerella forsythia. *Periodontol 2000* 54, 106–116. doi: 10.1111/j.1600-0757.2009.00332.x

Takeuchi, H., Sasaki, N., Yamaga, S., Kuboniwa, M., Matsusaki, M., and Amano, A. (2019). Porphyromonas gingivalis induces penetration of lipopolysaccharide and peptidoglycan through the gingival epithelium via degradation of junctional adhesion molecule 1. *PLoS Pathog* 15, e1008124. doi: 10.1371/journal.ppat.1008124

Tedesco, D., Thapa, M., Chin, C. Y., Ge, Y., Gong, M., Li, J., et al. (2018). Alterations in Intestinal Microbiota Lead to Production of Interleukin 17 by Intrahepatic γδ T-Cell Receptor-Positive Cells and Pathogenesis of Cholestatic Liver Disease. *Gastroenterology* 154, 2178–2193. doi: 10.1053/j.gastro.2018.02.019

Tonomura, S., Hattori, Y., Ishibashi, T., Ikeda, S., Noda, K., Chiba, T., et al. (2025). Oral Pathobiont Streptococcus Anginosus Is Enriched in the Gut of Stroke Patients and Predicts 2-Year Cardiovascular Outcome. *Circ J*. doi: 10.1253/circj.CJ-24-0872

Vonaesch, P., Araújo, J. R., Gody, J.-C., Mbecko, J.-R., Sanke, H., Andrianonimiadana, L., et al. (2022). Stunted children display ectopic small intestinal colonization by oral bacteria, which cause lipid malabsorption in experimental models. *Proc Natl Acad Sci U S A* 119, e2209589119. doi: 10.1073/pnas.2209589119

Wei, Y., Zhang, Y., Zhuang, Y., Tang, Y., Nie, H., Haung, Y., et al. (2024). Veillonella parvula acts as a pathobiont promoting the biofilm virulence and cariogenicity of Streptococcus mutans in adult severe caries. *Microbiol Spectr* 12, e0431823. doi: 10.1128/spectrum.04318-23

Wu, B. G., Sulaiman, I., Tsay, J.-C. J., Perez, L., Franca, B., Li, Y., et al. (2021). Episodic Aspiration with Oral Commensals Induces a MyD88-dependent, Pulmonary T-Helper Cell Type 17 Response that Mitigates Susceptibility to Streptococcus pneumoniae. *Am J Respir Crit Care Med* 203, 1099–1111. doi: 10.1164/rccm.202005-1596OC

Wu, L., Shi, R., Bai, H., Wang, X., Wei, J., Liu, C., et al. (2022a). Porphyromonas gingivalis Induces Increases in Branched-Chain Amino Acid Levels and Exacerbates Liver Injury Through livh/livk. *Front Cell Infect Microbiol* 12, 776996. doi: 10.3389/fcimb.2022.776996

Wu, Z., Mehrabi Nasab, E., Arora, P., and Athari, S. S. (2022b). Study effect of probiotics and prebiotics on treatment of OVA-LPS-induced of allergic asthma inflammation and pneumonia by regulating the TLR4/NF-kB signaling pathway. *J Transl Med* 20, 130. doi: 10.1186/s12967-022-03337-3

Xu, B., and Han, Y. W. (2022). Oral bacteria, oral health, and adverse pregnancy outcomes. *Periodontol 2000* 89, 181–189. doi: 10.1111/prd.12436

Xu, H., Sobue, T., Bertolini, M., Thompson, A., and Dongari-Bagtzoglou, A. (2016). Streptococcus oralis and Candida albicans Synergistically Activate μ-Calpain to Degrade E-cadherin From Oral Epithelial Junctions. *J Infect Dis* 214, 925–934. doi: 10.1093/infdis/jiw201

Zhang, L., Zhang, D., Liu, C., Tang, B., Cui, Y., Guo, D., et al. (2024). Outer Membrane Vesicles Derived From Fusobacterium nucleatum Trigger Periodontitis Through Host Overimmunity. *Adv Sci (Weinh)* 11, e2400882. doi: 10.1002/advs.202400882

Zhang, X., Cheng, S., Chen, S., Wang, Q., Zhou, J., Wang, H., et al. (2025). Periodontitis-associated Fusobacterium nucleatum promotes ulcerative colitis by ferroptosis-mediated gut barrier disruption. *NPJ Biofilms Microbiomes* 11, 155. doi: 10.1038/s41522-025-00763-1

Zhou, C.-B., Zhao, L.-C., Qin, Y., Yu, J., Li, W., Feng, Q., et al. (2026). Streptococcus anginosus-derived methionine promotes gastric cancer progression. *Gut*, gutjnl-2025-336966. doi: 10.1136/gutjnl-2025-336966
